# Phospholipase D3 degrades mitochondrial DNA to regulate nucleotide signaling and APP metabolism

**DOI:** 10.1038/s41467-023-38501-w

**Published:** 2023-05-24

**Authors:** Zoë P. Van Acker, Anika Perdok, Ruben Hellemans, Katherine North, Inge Vorsters, Cedric Cappel, Jonas Dehairs, Johannes V. Swinnen, Ragna Sannerud, Marine Bretou, Markus Damme, Wim Annaert

**Affiliations:** 1 Laboratory for Membrane Trafficking, VIB Center for Brain & Disease Research, Herestraat 49, box 602, Leuven, Belgium; 2 Department of Neurosciences, KU Leuven, Herestraat 49, box 602, Leuven, Belgium; 3 Laboratory for Molecular Cell Biology and Transgenic Research, Institute of Biochemistry, Christian-Albrechts-University Kiel, Otto-Hahn-Platz 9, Kiel, Germany; 4 Laboratory of Lipid Metabolism & Cancer, Department of Oncology, KU Leuven, B−3000 Leuven, Belgium

**Keywords:** Mitophagy, Mechanisms of disease, Lysosomes, Cellular neuroscience

## Abstract

Phospholipase D3 (PLD3) polymorphisms are linked to late-onset Alzheimer’s disease (LOAD). Being a lysosomal 5’-3’ exonuclease, its neuronal substrates remained unknown as well as how a defective lysosomal nucleotide catabolism connects to AD-proteinopathy. We identified mitochondrial DNA (mtDNA) as a major physiological substrate and show its manifest build-up in lysosomes of PLD3-defective cells. mtDNA accretion creates a degradative (proteolytic) bottleneck that presents at the ultrastructural level as a marked abundance of multilamellar bodies, often containing mitochondrial remnants, which correlates with increased PINK1-dependent mitophagy. Lysosomal leakage of mtDNA to the cytosol activates cGAS–STING signaling that upregulates autophagy and induces amyloid precursor C-terminal fragment (APP-CTF) and cholesterol accumulation. STING inhibition largely normalizes APP-CTF levels, whereas an APP knockout in PLD3-deficient backgrounds lowers STING activation and normalizes cholesterol biosynthesis. Collectively, we demonstrate molecular cross-talks through feedforward loops between lysosomal nucleotide turnover, cGAS-STING and APP metabolism that, when dysregulated, result in neuronal endolysosomal demise as observed in LOAD.

## Abstract (web summary)

Phospholipase D3 polymorphisms are linked to late-onset Alzheimer’s disease but the mechanisms are not understood. Van Acker and colleagues show that Phospholipase D3 processes mitochondrial DNA in lysosomes to maintain lysosomal homeostasis and proper degradation of the amyloid precursor protein.

## Introduction

Neurons rely on lysosomal homeostasis for the removal of obsolete organelles and protein aggregates, but also for endosomal protein and lipid turnover and cargo recycling. Unsurprisingly, endolysosomal abnormalities emerge in early, preclinical stages of neurodegenerative diseases^1–3^. In Alzheimer’s disease (AD), in particular, the amyloidogenic processing of the amyloid precursor protein (APP) intimately revolves in and around the endolysosomal system^4,5^. Toxic amyloid-β (Aβ) peptides arise in endolysosomes well before extracellular amyloid plaque deposition^6^. Late endosomes and lysosomes meet the high Aβ concentration, redox requirements, and acidic environment for intracellular Aβ to form high molecular weight species that can progress into amyloid fibrils and plaques; features that are not readily met in the extracellular space^7–10^. Moreover, endosomes and lysosomes get significantly enlarged in neurons affected by AD, as demonstrated in in vitro cellular^11–13^ and in vivo murine AD models^14,15^ as well as in the brains of AD patients^16^. This enlargement underscores a stressed endocytic pathway activity and/or maturation.

Genetics further support a causal role for endolysosomal dysfunction in AD. Genome-wide association studies identified a few dozen risk genes for late-onset AD (LOAD) that cohere chiefly to three biological pathways; the innate immune response, cholesterol metabolism, and endolysosomal transport regulation^17^. In turn, all three pathways are closely linked to APP metabolism and Aβ clearance^3,18–20^. Besides the many ‘endocytic’ genes that function in endocytosis, cargo sorting, and recycling, *PLD3* encodes a bona fide lysosomal protein that is highly expressed in brain neurons^21^. This makes PLD3 one of the few risk factors that potentially directly links neuronal lysosomal dysfunction to LOAD initiation and progression.

The *PLD3* gene encodes the phospholipase D3 (PLD3) protein that primarily localizes to lysosomes^22,23^. It is a member of the PLD superfamily that is characterized by the occurrence of two highly conserved HxKxxxxD motifs that are critical for phospholipase-D activity^24^. The substitution of any amino acid in either HxKxxxxD motif leads to a loss of enzymatic function^24^. In this regard, unlike other members of the PLD family (PLD1 and −2), PLD3 has been suggested to lack phospholipase-D activity due to its second HxKxxxxD motif containing a conserved glutamate to aspartate substitution^25^. In accordance, a study using shotgun lipidomics on PLD3 knockout brain lysates did not retrieve clear alterations in phosphatidic acid species or phosphatidylcholine levels^26^. More recently, however, Nackenoff and colleagues countered this by demonstrating phospholipase-D activity for PLD3^27^. Awaiting further characterization of these inconsistencies, PLD3 and its close relative PLD4 have been shown to function as redundant 5’−3’ exonucleases; for which a single copy of the HxKxxxxD motif suffices^28–31^. In the mouse peripheral immune system, they help to keep the responses of nucleic acid-sensing toll-like receptors (TLR) in check^30,31^. Mice deficient for both PLD3 and PLD4 go into an inflammatory state that is already lethal early in life^30^. Where PLD4 shows an expression mainly linked to (peripheral) immune cells, PLD3 expression is selectively enriched in neuronal populations^21^, with those in the frontal-temporal-occipital cortices and the hippocampus showing the highest levels; regions of the brain characteristically vulnerable to AD pathology. Thus as PLD4 cannot compensate for PLD3 in neurons, they are expected to be more vulnerable to PLD3 loss-of-function. Indeed, PLD3 deficiency affects lysosomal morphology in vivo and in vitro^22,32,33^. However, thus far, no progress has been made in the understanding of the substrates nor the impact of PLD3 lysosomal (dys)function in neurons.

Of the different coding single nucleotide polymorphisms (SNPs) identified in *PLD3*, the V232M SNP is the most clinically represented^33,34^. It is not detected in the cognitively healthy elderly and worsens story recall as well as visual learning. Moreover, memory decline in AD patients and carriers with this SNP shows a trend toward a faster disease progression^33,34^. In general, 7.99% of AD cases presents with a PLD3 variant, a value twice as high as in a cognitively healthy control group^33^. Of note, not all genetic studies did identify a significant association of PLD3 variants with LOAD^35^. Nonetheless, even in the absence of *PLD3* SNPs, AD pathology causes epigenetic disturbances in the promoters of PLD3^36^. This explains at least in part the independent observations that neuronal PLD3 level become almost halved during the course of AD progression^33,37–40^, suggesting a wider impact in sporadic disease.

Knockdown and LOAD-linked variants of PLD3 have been associated with dysregulated APP processing and an increase in extracellular Aβ, while PLD3 overexpression was shown to decrease APP full length and Aβ levels^22,33,41^. PLD3 immunoreactivity was further shown to amass on neuritic plaques in AD brains^27,37^. In contrast, however, an in vivo study on 3 months old PLD3^−/−^ mice crossed with the APP^NL-G-F/NL-G-F^ knock-in failed to detect alterations in APP proteolytic fragments in brain tissue^32^, requiring further research.

In this study, we used CRISPR/Cas9 gene editing to generate PLD3 knockout (KO) SH-SY5Y cells that were subsequently stably rescued with wild-type PLD3 and LOAD-linked coding variants. We conclusively demonstrate that CpG-rich mitochondrial DNA (mtDNA) is the main PLD3 substrate in neuronal cells and that an altered lysosomal 5′−3′ exonuclease activity, through PLD3 KO or coding variants, dramatically impacts lysosomal homeostasis, integrity, and proteolytic capacity, including a defect in mitophagy. The failure of lysosomes to properly degrade ss/mtDNA activates downstream signaling, including toll-like receptor9 (TLR9) and the cyclic GMP–AMP synthase (cGAS)-stimulator of interferon genes (STING) pathway, the latter of which in turn affects APP proteolysis. Hence, improper lysosomal nucleotide handling through altered PLD3 functioning connects lysosomal dyshomeostasis with mitophagy, altered cholesterol metabolism, and APP processing; all reminiscent features of AD neuropathogenesis.

## Results

### An altered PLD3 exonuclease activity affects lysosomal (mt)DNA content

**Fig. 1:**
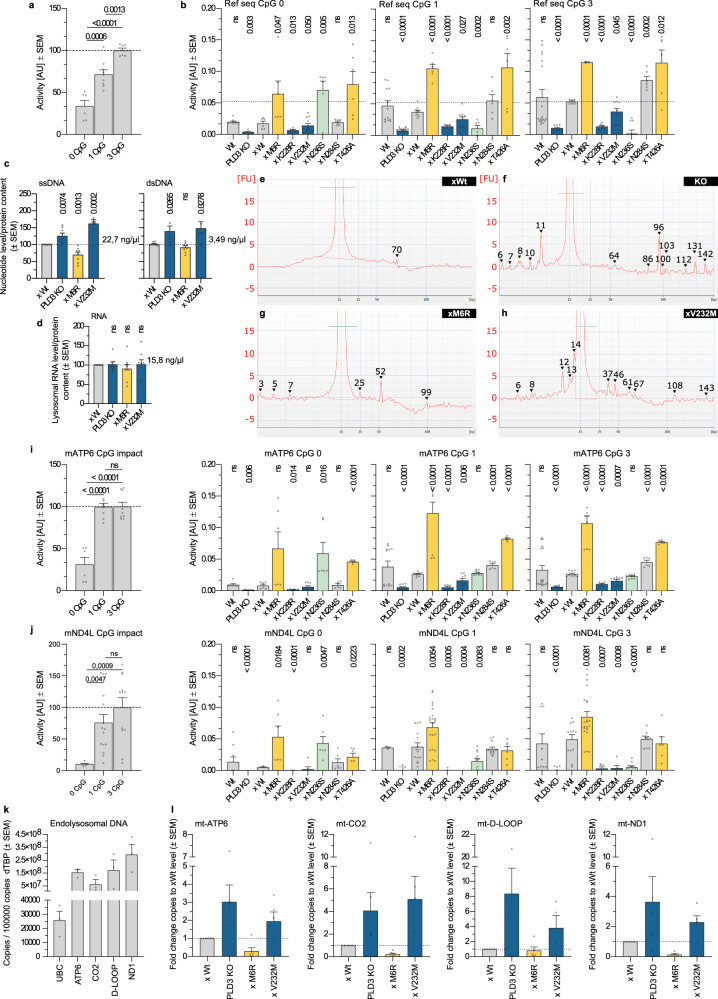
PLD3’s exonuclease function impacts the endolysosomal nucleotide content. **a**, **b** End-labeled fluorescence-quenched oligonucleotide (EFQO) assay for exonuclease activity analysis. **a** Lysates (500 ng/µL) of PLD3 KO cells rescued with wild-type PLD3 were incubated at 37 °C (pH 5) with a 30 nucleotide long oligo of a random sequence that was taken from ref. ^30^. The impact of the CpG-content was investigated by reducing the original 3xCpG content; CpGs in the sequence were adapted to TpG. The mean values of biologically independent samples are represented in the bar graphs. Statistical analysis was performed with two-tailed, unpaired *t*-tests. **b** Analysis of the different SNP lines as in **a**. **c**, **d** Lysosomal nucleotide content as measured on a Qubit fluorometer. Statistical analysis was performed with two-tailed, unpaired *t*-tests. (*n* = 8). **e**–**h** Electropherograms of the endolysosomal DNA content of **e** xWt, **f** PLD3 KO, **g** xM6R, and **h** xV232M cells. Standard: the small molecular weight marker measures 15 bp. **f** The peak of 96 bp amounts to 0.81 ng/µl, while **h** the 37 and 46 bp peaks amount to 0.09 and 0.07 ng/µl, respectively. **i**, **j** EFQO analysis on 30 nucleotides long part of the **i** mATP-6 and **j** mND4L sequence, each of which contains three CpGs. Substrates’ original 3xCpG content was reduced by T substitution and the assay was run in triplicate. Statistical analysis was performed with two-tailed, unpaired *t*-tests. **k**, **l** Quantitative PCR levels of mitochondrial mtDNA genes (ATP6, CO2, D-Loop, and ND1) and nuclear UBC in total DNA extracts from endolysosomal isolates (**k**: *n* = 3, **l**: *n* for xV232M = 5 and *n* for xWt/PLD3 KO/xM6R = 6 biological repeats). Source data are provided as a Source Data file.

Given PLD3 is highly enriched in neuronal lysosomes^22,23^ and given its connection to LOAD^33,34^, we opted to study its role in SH-SY5Y neuroblastoma cells (Fig. S1a–f). We generated two independent KO lines using CRISPR/Cas9 gene editing (Fig. S1g). These were used to subsequently create stable rescue lines, expressing either wild-type (Wt) PLD3 or coding SNP variants, including M6R, K228R, V232M, N236S, N284S, and T426A (Fig. S1a, g, h). PLD3 is synthetized as a type II membrane protein in the endoplasmic reticulum (ER) and, upon arrival in the lysosome, proteolytically cleaved, releasing the luminal domain of ~55 kDa that is considered to be the mature form^23^. We first tested whether the maturation of PLD3 is affected by SNP variants. All variants largely processed full-length PLD3 to the ~55 kDa luminal fragment that was strongly enriched in purified lysosomes (Fig. S1i), indicating that these mutations did not significantly affect PLD3 trafficking and maturation. We next investigated PLD3’s exonuclease activity, using an End-labeled Fluorescence-Quenched Oligonucleotide (EFQO) assay (Fig. 1a, b). While the assay was previously used with a 30-nucleotide-long oligo of a random sequence^29,30^, we now extended this analysis towards CpG-content preference for which CpG sites were substituted with a thymine. A strong positive correlation was noticed between PLD3 exonuclease efficiency and CpG-content; with a particular reduced efficiency when the substrate contains no CpGs (Fig. 1a). A similar CpG-rich preference was seen for all variants, except the N236S variant that showed a high activity towards the CpG-null sequence (Fig. 1b, green). In general, the SNPs clustered around the PDE1 domain of PLD3 showed decreased exonuclease activities (Fig. 1b, blue), while the reverse was seen for those SNPs located at the termini (Fig. 1b, yellow). Hence, for more elaborate experiments, we chose to compare PLD3^−/−^ SH-SY5Y cells with PLD3^−/−^ xWt, xM6R, and xV232M rescued lines. Reliably representing the clinical situation, these four lines provide a range of exonuclease activities, going from absent (PLD3^−/−^) to a decreased (xV232M) and increased (xM6R) activity as compared to Wt levels.

Given PLD3 preferentially degrades CpG-rich ssDNA, it is likely that its deficiency or change in activity may directly affect and/or accelerate the build-up of (certain CpG-rich) ssDNA ligands. We first analyzed magnetically purified lysosomes for the presence of ssDNA, dsDNA, and RNA, using a Qubit 4 Fluorometer (Fig. 1c, d). Endogenous mean values for Wt reached 22.7 ng/µl for ssDNA, 3.49 ng/µl for dsDNA, and 15.8 ng/µl for RNA. Interestingly, the DNA nucleotide content followed the exonuclease activity profile, with lysosomes from PLD3^−/−^ and xV232M cells showing an accretion of DNA (Fig. 1c). The enhanced exonuclease activity of the M6R variant resulted in lower lysosomal ssDNA and dsDNA levels. No apparent effect was seen on the lysosomal RNA level (Fig. 1d), supporting that RNA is not a major endogenous substrate. We next performed microfluidic electrophoresis to obtain a size-profile of the accumulating DNA species (Fig. 1e–h). Whereas xWt rescued cells exhibited a continuum of species between ~5–70 bp without apparent peaks (Fig. 1e), PLD3^−/−^ and xV232M rescued cells displayed a pattern of peaks (Fig. 1f, h). The M6R SNP gave a generally low signal, corresponding to its increased exonuclease activity, though some peaks could still be observed (Fig. 1g). Hence, these data point to an additional level of substrate specificity.

Macrophages of PLD3^−/−^ mice show an exacerbated cellular response to exogenous CpG agonists of TLR9^30^. Mitochondria are evolutionary endosymbionts with a bacterial origin and the mitochondrial genome similarly contains high levels of inflammatogenic unmethylated CpG motifs^42^. The latter may be particularly relevant to neurons with their high energetic activity and associated high mitochondrial turnover^43^. Hence, we hypothesized that mtDNA might be a major and constant source of PLD3 substrates, including under basal conditions. To test this, we extended our EFQO assays to oligonucleotide sequences of mitochondrial ATP synthase 6 (mATP-6) and NADH-ubiquinone oxidoreductase chain 4 L (mND4L). PLD3 readily degraded mitochondrial sequences (Fig. 1i, j). As with the random sequence (Fig. 1a), kinetics and/or affinity dropped with lowered CpG-content. PLD3 variants around the PDE1 domain also displayed lowered exonuclease activities, while the reverse was seen for the terminal SNPs, M6R, and T426A (Fig. 1i, j). We, therefore, investigated whether we could detect mtDNA in extracts of purified lysosomes. As shown in Fig. 1k, the lysosomal DNA content of Wt-rescued SH-SY5Y cells comprised mainly of mtDNA. Values of up to four mtDNA genes strongly exceeded those of nuclear transcripts. In line with the total DNA content of purified endolysosomes (Fig. 1c) and PLD3’s preference towards CpG motifs in mtDNA sequences (Fig. 1i, j), PLD3 exonuclease activity was reflected in the mtDNA content of purified endolysosomes (Fig. 1l). These data strongly support that mtDNA is a major endogenous substrate for PLD3 in lysosomes.

### A balanced PLD3 exonuclease activity is required to maintain lysosomal homeostasis

**Fig. 2:**
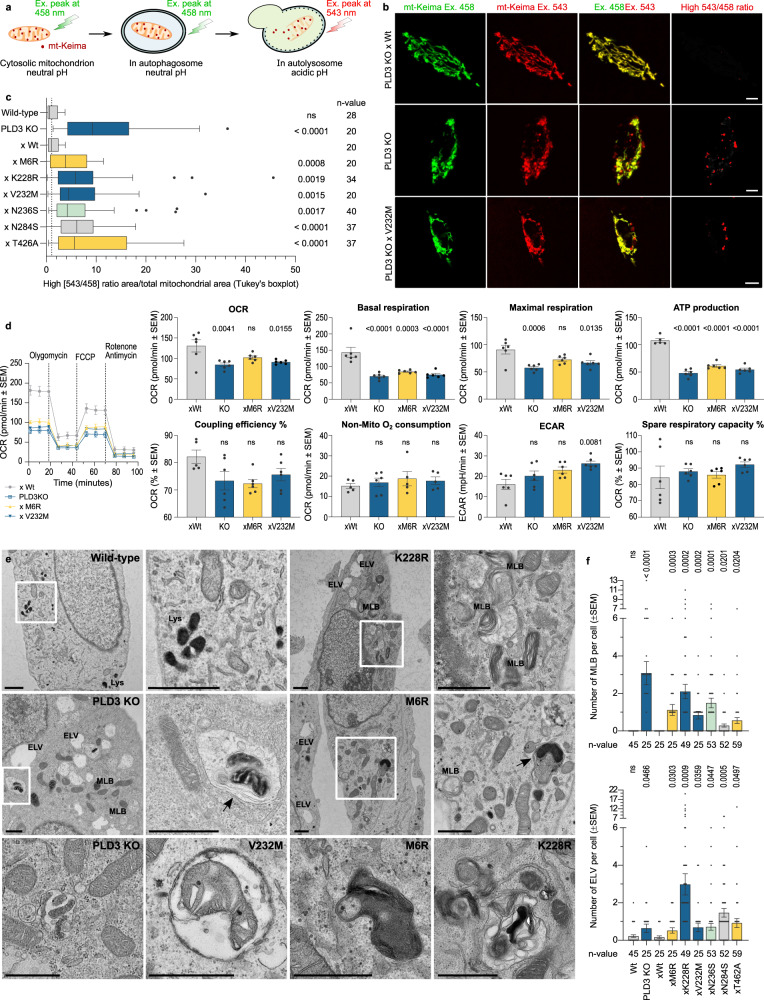
Mitochondrial fitness and clearance are disrupted by PLD3 dysfunction. **a** Schematic representation of Mt-Keima, showing a pH-dependent shift in fluorescence excitation that can be imaged in two channels. **b** Representative confocal images of SH-SY5Y PLD3 cell models. Scale bar = 5 µm. **c** Tukey’s boxplot (first to third quartile box with the median as a horizontal line) showing the index of mitophagy, which was calculated as the parameter: high [543/458] ratio area/total mitochondrial area (*n* = 20–40). Two-tailed, unpaired *t*-tests were used for statistical testing. Differences with the wild-type rescue condition are indicated. **d** Mitochondrial bioenergetic profiles were obtained with the Seahorse XFp Cell Mito Stress test, including oxygen consumption rate (OCR) and extracellular acidification rate (ECAR). An ordinary one-way ANOVA with Bonferroni’s multiple comparisons test was used for statistical testing (*n* = 6; columns show mean values ± SEM). **e** Representative EM images of PLD3 KO and SNP variant-rescued SH-SY5Y cells showing an aberrant accumulation of degradative organelles, including MLBs and autophagosomes/autophagolysosomes, often with remnants of mitochondria. ELV electron-lucent vesicles, MLB multilamellar bodies. Scale bar is 1 µm. **f** Quantification of the number of MLB and ELV per cell in two PLD3 clonal knockout lines, of which results were pooled together. Statistical significance was tested in comparison to the wild-type rescue values using a two-tailed, unpaired *t*-test (*n* = 25–59). Source data are provided as a Source Data file.

**Fig. 3:**
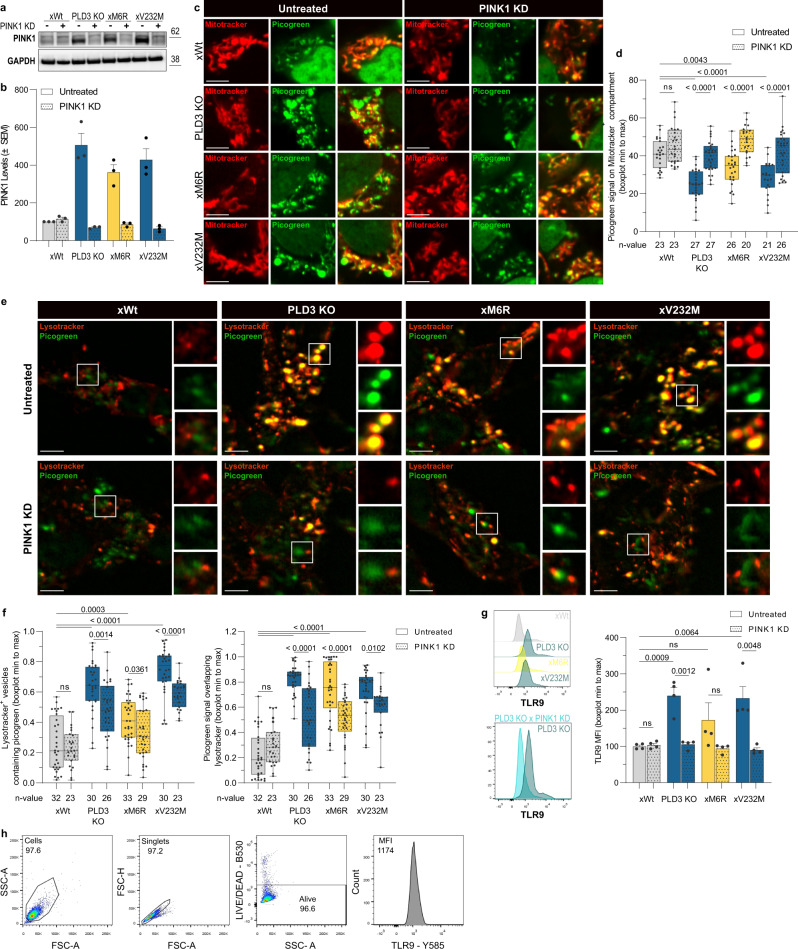
PINK1-linked mitophagy delivers PLD3 substrates to lysosomes that cause a DNA build-up upon PLD3 dysfunction. **a** Protein levels of PINK1, quantified in (**b**). PINK1 knockdown (KD) reduced PINK1 levels to baseline 24 h after transfection (*n* = 3; mean values ± SEM). **c** Representative images of mitotracker (mitochondria, red) — picogreen (ds/mtDNA, green) co-localizations. **d** Min to max boxplots (median as the center, the box extends from the 25th to 75th percentiles, and whiskers range from the smallest to the greatest value) depict the fraction of picogreen overlapping with mitotracker signal (*n* = 20–27). **e** Representative images of lysotracker (acidic compartment, red) — picogreen (ds/mtDNA, green) co-localizations. Scale bar = 5 µm in all cases. **f** Min to max boxplots show the quantification of the fraction of overlap between the lysotracker and picogreen signal, using Manders’ coefficients (*n* = 23–33). **g** Intracellular flow cytometry of TLR9 levels. The stopping gate was set at 10,000 events (*n* = 4). All statistical differences were calculated using unpaired *t*-tests as indicated. **h** Gating strategy of panel **g**. Source data are provided as a Source Data file.

The correlation of PLD3 exonuclease activity with levels of lysosomal-localized mtDNA hinted at a similarly altered mitophagic rate and/or reduced lysosomal degradative capacity. To test this, we used the mitophagy reporter mKeima-Red-Mito-7^44^. SH-SY5Y cells lacking PLD3 or harboring an SNP displayed an increased mitophagy level (Fig. 2a–c), which was as well reflected in increased PINK1 protein levels (Fig. 3a, b). We next checked whether mitochondrial fitness would be affected, using a Seahorse metabolic analysis. PLD3^−/−^ as well as xM6R and xV232M rescued SH-SY5Y cells, all exhibited a significant decrease in their basal respiration, maximal respiration, and ATP production compared to xWt rescued cells (Fig. 2d). These defects in mitochondrial metabolism are conform the observed increased number of mitochondria with a low membrane potential, measured independently using the JC1 probe (Fig. S2a–d). Importantly, an increased mitophagy index and decreased mitochondrial fitness were observed for all PLD3 SNP variants, irrespective of whether they have a decreased or increased exonuclease activity. Thus, lysosomes require a homeostatic regulation of exonuclease activity, the disruption of which causes a defective mitochondrial turnover.

Our findings also suggested that an altered exonuclease activity indifferently results in a defective or over-solicited lysosomal degradative capacity. In support, transmission electron microscopy (TEM) analysis demonstrated a striking accumulation of multilamellar bodies (MLBs) in PLD3^−/−^ and SNP-variant lines (Figs. 2e, f and S2e). MLBs are stalled autolysosomes that fail to degrade/remove cellular organelles through autophagy, in particular mitochondria, leading to a strong build-up of lipid/membrane whorls^45–47^. TEM further indicated the degradative vesicles to be of autophagic origin, with some of the vesicles still containing a double membrane, or autolysosomes, given their darker appearance and degradative material content (Fig. 2e). These data point to mitophagy being a key step in the lysosomal pathology that is linked to PLD3 dysfunction. To substantiate this, we checked whether a PINK1 knockdown (KD), using siRNA, would rescue the lysosomal DNA build-up and pathology. In xWt PLD3 expressing cells, picogreen-stained mtDNA is mostly overlapping with mitochondrial Mitotracker, while strongly shifting to Lysotracker-positive lysosomes in PLD3 KO and SNP cells; underscoring increased mitophagy (Fig. 3c–f). A subsequent PINK1 KD rescues the effect partially; lowering the picogreen-labeled mtDNA levels in lysosomes (Fig. 3e, f) and increasing the overlap again with mitochondria (Fig. 3c, d). To see whether such a lowered supply of PLD3 substrates would have a functional effect in lysosomes, we measured TLR9 levels. TLR9 levels rapidly respond upon CpG-DNA presence in lysosomes and only have a half-life of 8 h^48^, making them ideal to functionally assessing the lysosomal DNA content. In accordance with the picogreen stainings, PLD3 KO and SNP cells show increased TLR9 levels that get significantly reduced after 24 h of PINK KD (Fig. 3g).

**Fig. 4:**
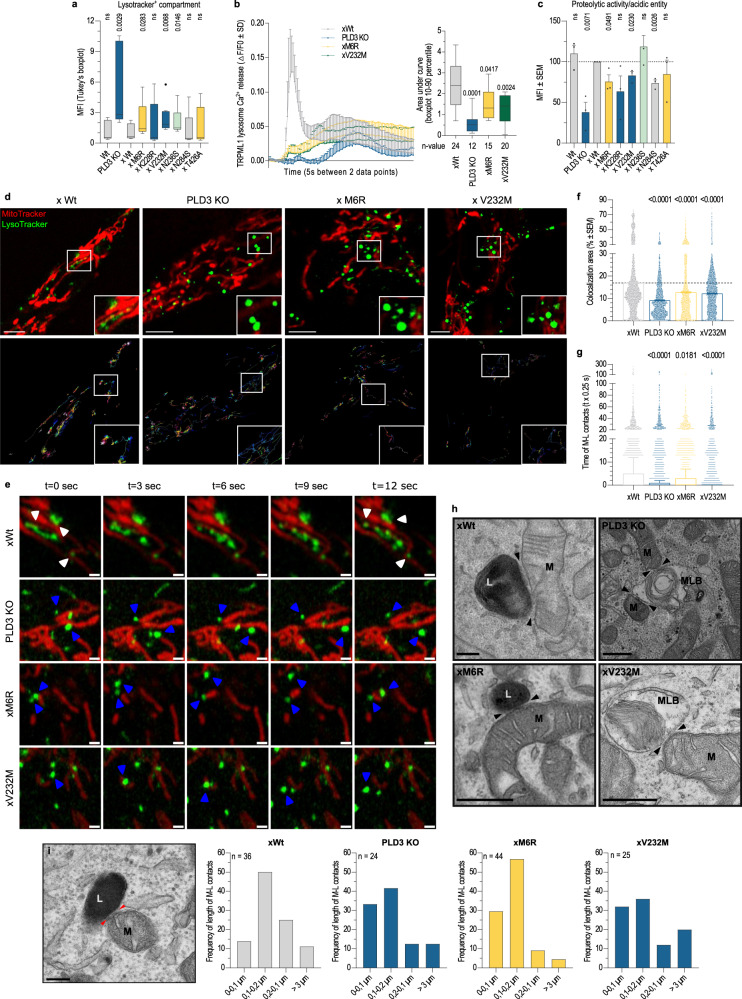
PLD3-linked mitophagic problems are routed in a dysfunctional EL catabolic activity and reinforced by less stable lysosome-mitochondria membrane contact-site interactions. **a** Flow-cytometric analysis of the acidic compartment, stained with 50 nM LysoTracker green (Tukey’s boxplot—first to third quartile box with the median as a horizontal line, *n* = 11). **b** Lysosomal Ca^2+^ response elicited with 20 µM of TRPML1 activator ML-SA1 was measured in Fura-2-AM loaded cells. Main panel: mean lysosomal Ca^2+^ response with SD error bars. Inset: quantification of individual Ca^2+^ responses (10–90 percentile box, showing the median as center and whiskers at the tenth and ninetieth percentiles; *n* = 12–24; two-tailed, unpaired *t*-tests with xWt condition). **c** Flow-cytometric analysis of the acidic compartment, stained with 10 µg/ml DQ-Red BSA and 50 nM LysoTracker green (*n* = 3). Two-tailed, unpaired *t*-testing was used for statistical testing with the wild-type rescue condition as a baseline. **d** Representative images of LE-mitochondria co-localizations (upper panel) and track dynamics (lower panel). Scale bar is 5 µm. **e** Lysosome-mitochondria contact tethering. Airyscan time-lapse images of stable (white arrow) and more transient (blue arrow) contacts in live SH-SY5Y cells. **f** Bar graphs showing the mean percentage overlap between lysosomes and mitochondria and **g** Tukey’s boxplot (first to third quartile box with the median as a horizontal line) depicting their contact duration. **f**, **g** Two-tailed, unpaired *t*-tests were used for statistical testing (individual contacts analyzed in *n* = 15, 16, 17, and 16 cells —xWt, PLD3 KO, xM6R, and xV232M— over *t* = 125 s). **h** Characteristic electron microscopy images of close contacts between mitochondria (M) and degradative vesicles, i.e., lysosomes (L) and multilamellar bodies (MLB). Scale bar = 500 nm. Two independent sample preps from two PLD3 clonal lines were analyzed per genotype. **i** Quantification of the fraction of contacts with a specific length range (*n* = 25–55); the length of which was measured as indicated by the red arrows on the representative image. Scale bar = 200 nm. Source data are provided as a Source Data file.

In addition to MLBs, an altered PLD3 function caused electron-lucent vesicles (ELVs), containing no or few intraluminal vesicles, to accumulate (Figs. 2e, f and S2e). These ELVs may reflect immature multivesicular bodies, suggesting a broader derailed endolysosomal system in PLD3^−/−^ or SNP-expressing cells. We, therefore, explored endolysosomal functions in more detail. The observed accumulation of degradative organelles correlated with a significant increase in LAMP1-immunoreactivity (Fig. S3a). Concomitantly, cells showed a significant increase in the Lysotracker-positive area (Fig. 4a), however, without the lysosomal pH being markedly affected (Fig. S2f). Instead, TRMPL1-induced lysosomal Ca^2+^ release was significantly decreased in PLD3^−/−^ and SNP variant rescued cells (Fig. 4b). Other hallmarks of endolysosomal dysfunction included a decreased processing to active/mature Cathepsin-D (Fig. S3a) and a decreased dequenching of LE/Lys-localized DQ-BSA (Fig. 4c), denoting a general decrease in lysosomal degradative capacity. Only in the case of xN236S-rescued cells, DQ-BSA dequenching was comparable to PLD3 Wt cells. This SNP variant has shifted its exonuclease activity chiefly to CpG-null nucleotides, but how such changes may differently affect endolysosomal functions remains to be further investigated.

### Lysosomal dyshomeostasis is echoed in an altered lipid composition and cholesterol accumulation

The observed endolysosomal defects caused by an altered PLD3 function could further underlie downstream changes in lipid metabolism and composition of endolysosomes. To investigate this, we compared lipid profiles of postnuclear supernatants (PNS) and lysosomes magnetically isolated from PLD3^−/−^, xM6R, and xV232M SH-SY5Y cells with those of xWt rescued cells. While overall, no differences in lipid unsaturation and carbon lengths were noted (Fig. S3b, c), the lysosomal fraction of storage lipids was increased two- to three-fold upon PLD3 dysfunction (Fig. S3d). The build-up is primarily attributed to the marked increase in cholesterol esters (CE, Fig. S3e, g and Supplementary Data), including CE 20:0, 20:5, 22:5, and 22:6 (Fig. S3g). Concomitantly, endolysosomes showed an increase in cholesterol content (Fig. S3f). Storage lipids like triacylglycerol strongly decreased in PLD3^−/−^ and SNP-variant rescued cells. Hence, increased CE, but not other storage lipids, may corroborate the observed accumulation of MLBs (Fig. 2f), as they contribute to lipid storage and secretion^46^.

Although some lipid classes did not exhibit a gross impact of PLD3 dysfunction, a more detailed volcano plot analysis did for specific species within, e.g., for hexosylceramides (Hexcer) d18:1/22:0, d18:1/22:2 and d18:1/22:3 (Fig. S3e, g). HexCers are structural building blocks for cell membranes and lipid rafts. Monoacylglyceride (MG) levels got overall reduced upon PLD3 depletion or in SNP-variant expressing cells (Fig. S3e, g). In particular, levels of MG 20:0, 20:1, 20:3, 20:4, 22:1 and 22:4 were decreased (Fig. S3e, g). MGs have been linked to mitochondrial metabolism, oxidative stress, and cellular senescence^49^. In addition, PLD3^−/−^ as well as SNP-variant cells, showed particular decreases in their lysosomal levels of both phosphatidylglycerol and phosphatidylethanolamine (PE; S3e). Notably, PE is a key phospholipid for mitochondrial respiratory function^50^, which we showed to be compromised in these cells (Fig. 2d).

### PLD3 deficiency and SNP variants destabilize lysosome-mitochondria contact sites

Organelles like lysosomes and mitochondria rely on membrane contact sites (MCSs) for the exchange of phospholipids, metabolites, and ions, and, as such, tightly regulate these processes^51^. For one, the lysosomal cholesterol environment influences protein tethering interactions^51^, of which the resulting alteration in close contacts has been described to affect the phospholipid and, in particular, cholesterol egress to mitochondria^52^. In addition, the mitochondrial Ca^2+^ flow gets regulated by the lysosomal TRPML1 Ca^2+^ channel at mitochondria-lysosome contacts^53^, of which responses are significantly affected in our PLD3 models (Fig. 4b). Hence, defects in lysosomal integrity and composition may impact this inter-organellar communication, thereby propagating or even accelerating dysfunction in other organelles such as mitochondria. Given we also observed the PLD3-linked lysosomal pathology to correlate with mitophagy and mitochondrial dysfunction, we reasoned that lysosome-mitochondria MCSs might be affected in PLD3^−/−^ and SNP-rescued SH-SY5Y cells. We investigated lysosome-mitochondria contact dynamics in Lyso- and Mitotracker co-stained cells, using high-speed Airyscan confocal live imaging. In accordance with a previous report^54^, 16.93 ± 0.18% of the acidic vesicles in PLD3^−/−^xWt rescued cells was in contact with mitochondria whensoever (Fig. 4d–f). This percentage was significantly decreased in PLD3^−/−^, xM6R and xV232M PLD3 models to 9.16 ± 0.08%, 12.73 ± 0.13%, and 12.23 ± 0.09%, respectively. The reduced number of lysosome-mitochondria MCSs was accompanied by a decreased contact duration between both organelles (Fig. 4g and Supplementary Videos 1–4) and a shift to shorter MCSs lengths (Fig. 4h, i). The majority (86,11%) of contacts in xWt cells showed a length of over 0.1 µm. Consistent with the decreased contact durations and overlap signals, the average length of lysosome-mitochondria contacts shifted towards smaller contact lengths (<0.1 µm) in PLD3^−/−^ or SNP-variant expressing cells (Fig. 4i).

### Altered PLD3 activity triggers downstream TLR9 signaling and cGAS-STING activation

**Fig. 5:**
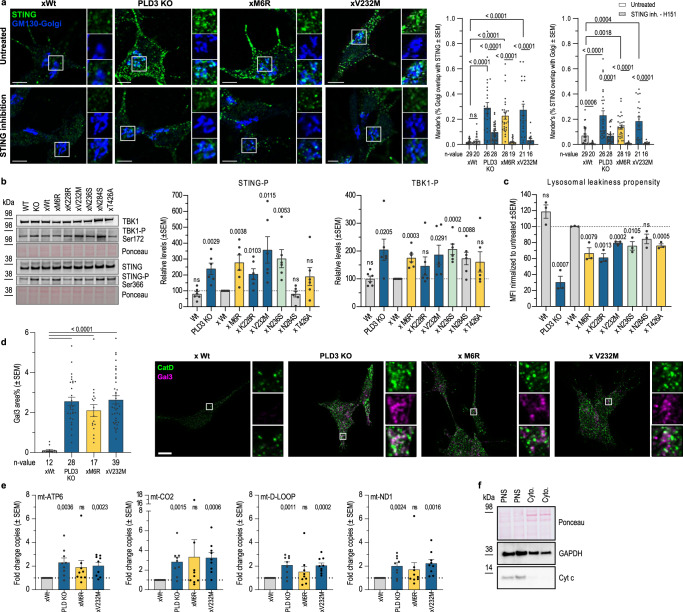
The PLD3-linked affected DNA catabolism impacts lysosomal integrity with subsequent STING activation. **a** STING’s activity-dependent clustering and relocalization to the Golgi complex was analyzed with the JaCoP2 Plugin of FIJI Image J and validated using a 5 h treatment with 1 µM H151 STING inhibitor. The scatter plots show Manders’ co-localization coefficients. Statistical significance was calculated using two-tailed, unpaired *t*-tests (*n* = 16–29). Scale bar = 5 µm. **b** Western blot analysis of STING signaling markers; i.e., TBK1 autophosphorylation at Ser172 and TBK1-induced STING phosphorylation of Ser366 of the STING C-terminal tail, which provides the docking site for IRF3 (*n* = 6). Two-tailed, unpaired *t*-testing was used for statistical testing with the wild-type rescue condition as a baseline. **c** Flow-cytometric analysis of the lysosomal permeability propensity, induced by the membrane destabilizing agent LLOMe (1 mM, 10 min). The stopping gate was set at 10,000 events (*n* = 3). Two-tailed, unpaired *t*-tests were used for statistical testing. **d** Quantification of Gal3 levels on confocal images (*n* = 12–39). Scale bar = 10 µm. Statistical significance was calculated using two-tailed, unpaired *t*-tests. **e** Quantitative PCR levels of mitochondrial mtDNA genes (ATP6, CO2, D-Loop, and ND1) in cytosolic extracts (mean of *n* = 9 biological repeats; unpaired *t*-testing shown to the xWt level), of which the purity was checked on **f** western blot. Source data are provided as a Source Data file.

As a defective lysosomal exonuclease activity leads to the build-up of, mainly, mtDNA fragments (Fig. 1k/l), this could trigger downstream nucleotide-sensing pathways; notably TLR9 (Fig. 3g) and cGAS-STING signaling. Firstly, and of note, nonimmune cells as neurons and SH-SY5Y cells also express TLR9 and respond to its stimulation^55^. TLR9 immunoreactivity was indeed upregulated in PLD3^−/−^ cells as well as in SNP variant-expressing cells (Fig. 3g and S4a–c) and relocated to the lysosomal membrane (Fig. S4d). Consequently, downstream inflammatory target genes, including IFN-α and TNF-α, got significantly increased (Fig. S4a); in agreement with previous findings^30^. Alternatively, the same inflammatory genes can become upregulated through the nucleotide-sensing cGAS-STING pathway, which senses cytosolic DNA. Upon binding to cGAMP, activated STING oligomerizes and translocates from the ER to the Golgi. Here it recruits TBK1 that undergoes autophosphorylation and phosphorylates STING. Firstly, STING partially relocated to the Golgi in PLD3^−/−^ and SNP-carrying cells (Fig. 5a); an activity-dependent clustering and redistribution that was reverted by a 5 h treatment with the STING inhibitor, H151 (Fig. 5a). Furthermore, and consistent with cGAS-STING activation, levels of phosphorylated STING (pSTING) and TBK1 (pTBK1) were significantly augmented in PLD3-affected cell lines (Fig. 5b). A stable rescue of PLD3^−/−^ SH-SY5Y cells with Wt PLD3 fully restored pSTING and pTBK1 levels, underscoring a direct connection to PLD3-mediated lysosomal exonuclease activity (Fig. 5b).

We have shown thus far that mtDNA accumulates in lysosomes purified from PLD3^−/−^ and xM6R and xV232M rescued cells (Fig. 1f–h, l). As cGAS senses cytosolic DNA to activate STING and mtDNA has been demonstrated to be an activator of STING^56^, we next investigated whether mtDNA could leak from lysosomes into the cytosol of cells with an altered PLD3 exonuclease activity. Firstly, cells were challenged with l-leucyl-l-leucine methyl ester hydrobromide (LLOMe)^57^. The decrease in Lysotracker signals in PLD3^−/−^ and all related SNP-variants reflected an increased vulnerability to LLOMe-induced leakiness (Figs. 5c and S4f). Secondly, PLD3^−/−^ and SNP-rescued cells recruited significantly more galectin-3 to lysosomes (Fig. 5d). Thus, in both stressed and unstressed conditions, PLD3^−/−^ and SNP-variant rescued cells displayed an increased lysosomal leakiness. To define whether mtDNA indeed leaked out to the cytosol, we used ultracentrifugation to isolate cytosolic fractions from the PNS of our different cell lines (Fig. 5f). Subsequent qPCR analysis showed a clear increase of all four mtDNA amplicons tested (ATP6, CO2, D-Loop and ND1) in the cytosol of PLD3^−/−^ and SNP-rescued cells (Fig. 5e). Collectively, these data demonstrate that an altered PLD3 activity renders lysosomes more leaky, at least in part contributing to the observed increase in cytosolic mtDNA levels that promote cGAS-STING activation.

### cGAS-STING activation-linked autophagy connects PLD3 dysfunction with APP metabolism

**Fig. 6:**
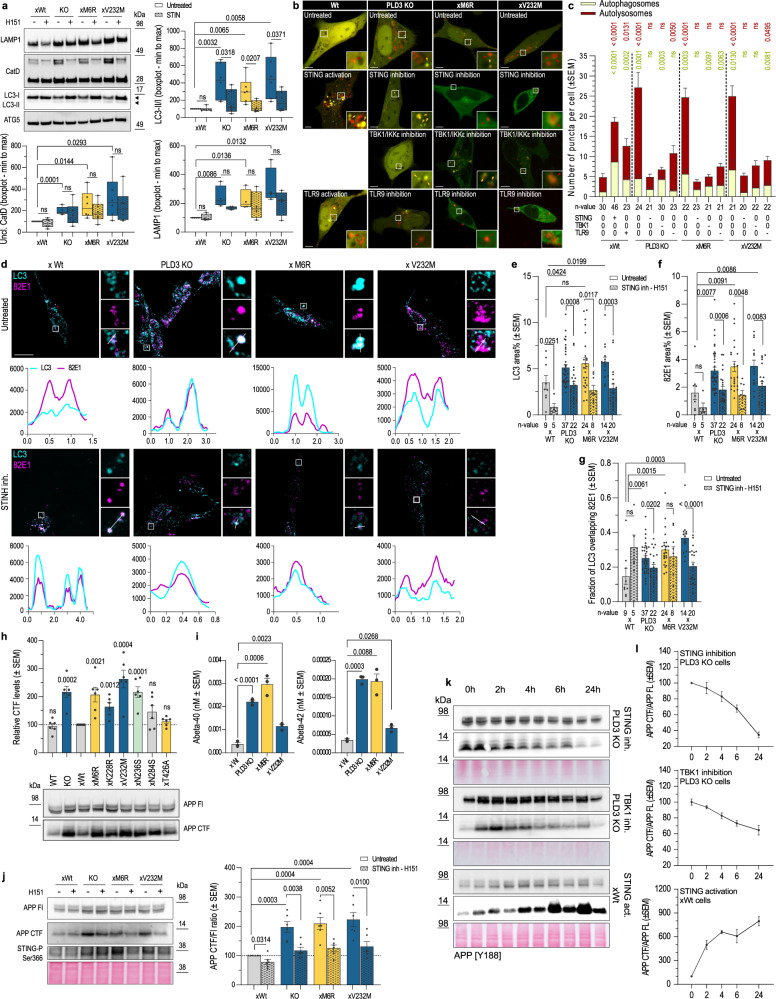
cGAS-STING activation-linked autophagy connects PLD3 dysfunction with APP metabolism. **a** Western blot analysis showing the rescue of 5 h H151-STING inhibition on PLD3-linked endolysosomal pathology. Statistical significance on boxplots (min to max; median as the center, the box extends from the 25th to 75th percentiles, and whiskers range from the smallest to the greatest value) was calculated using two-tailed, unpaired *t*-tests (*n* = 6). **b** Representative airyscan images of autophagosome/-lysosome puncta, using mCherry-GFP-LC3 in untreated SH-SY5Y or treated 5 h with 1 µM H151 STING inhibitor, 2 µg/mL cGAMP STING-ligand, 1 µM TBK1/IKKε inhibitor, or 2.5 µM TLR9-ligand or -inhibitor. Scale bar = 5 µm. **c** Quantification of b. Activation and inhibition are indicated as + and −, respectively. Two-tailed, unpaired *t*-tests with the xWt untreated baseline condition are indicated for both autophagosome and –lysosome counts (specific *n*-values are listed on the graph). **d** Up: representative images of PLD3 SH-SY5Y models stained for LC3 (cyan) and APP-CTF (82E1 antibody, magenta). Scale bar = 10 µm. Down: Colocalization analysis using line intensity profiles. **e**–**g** Quantification of d using Manders’ coefficient and area volume (*n* = 5–37). Statistical differences were calculated using two-tailed, unpaired *t*-tests. **h** Western blot showing APP fragment build-up. Statistical deviation from WT-rescue was calculated with unpaired *t*-tests (*n* = 6). **i** Secreted Aβ40 and −42 in 72-h-conditioned medium from SH-SY5Y (*n* = 3). Two-tailed, unpaired *t*-tests were used for statistical testing. **j** Left: a representative blot on the rescue effect of 5 h of STING inhibition on APP-CTF levels. Right: statistical significance was calculated using two-tailed, unpaired *t*-tests (*n* = 6). **k** STING activation induces APP-CTF increases in xWt cells, while its inhibition as well as of downstream TBK1, rescues PLD3-linked APP-CTF increases in PLD3 KO cells. **l** Quantifications of k (*n* cGAMP = 3, *n* H151 and TBK1 inh = 4). Source data are provided as a Source Data file.

Interestingly, STING activation has been linked to both autophagosome-lysosome fusion^58^ and autophagy initiation^59^. In addition, the latter can occur either through a non-canonical autophagy pathway, involving ATG5-12-16L1 complex activation by WIPI2, or through the canonical axis involving TBK1-ULK^59^. We, therefore, investigated whether autophagy would be affected in PLD3^−/−^ and SNP-variant expressing cells. Firstly, under basal conditions, the ratio of lipidated LC3II over LC3I was increased (Fig. 6a), consistent with the capacity of activated STING to induce LC3 lipidation^60^. Accordingly, the LC3^+^-compartment was significantly increased on confocal images (Fig. 6d, e). Transfection using the mCherry-GFP-LC3 autophagic flux reporter indicated a significant build-up of both autophagosomes (yellow in (Fig. 6b/c)) and autolysosomes (red in (Fig. 6b, c)) in PLD3^−/−^ and SNP-variant cells. Of note, the share of autolysosomes was far larger. Together with the reduced catabolic activity of lysosomes (Fig. 4c), this implies that not the fusion of autophagosomes with lysosomes but rather the lysosomal degradative capacity got significantly compromised. If downstream of hyperactivated STING, we argued that these defects could be reverted by inhibiting STING signaling. A 5 h treatment of PLD3^−/−^ or SNP-carrying cells with STING inhibitor significantly decreased LC3II/LC3I ratios and ameliorated LAMP1 levels as well as the maturation of cathepsin-D (Fig. 6a). This was accompanied by a significant decrease in the number of LC3-positive autophagosomes and autolysosomes (Fig. 6b, c, e). Of note, whereas H151 treatment more modestly decreased total cholesterol levels (Fig. S5a), (autophago)lysosomal membrane localized cholesterol decreased to near the levels in Wt-rescued cells (Fig. S5b, c). Together, these data underscore that the observed autophagy-lysosome defects are predominantly downstream of STING hyperactivation in PLD3^−/−^ and SNP-variant expressing cells. Inhibiting TBK1, a downstream target of STING activation, as well as TLR9 signaling, using the ODN-INH-18 Class B inhibitor, also reduced the number of LC3-positive autophagosomes and autolysosomes towards the baseline level (Fig. 6b, c). As a PINK1 knockdown lowered the levels of mtDNA in lysosomes (Fig. 3e, f), we wondered whether this would result in a likewise normalization of the autophagic flux. However, in contrast to STING and TLR9 inhibition, PINK1 knockdown resulted in a significant autophagosome build-up relative to a lowered autolysosome accumulation (Fig. S5e), in agreement with previous studies showing an increased rate of autophagosome formation^61^, endosomal trafficking problems and an increased cell death vulnerability in PINK1-deficient cells^62,63^.

We have shown that STING hyperactivation in PLD3^−/−^ and SNP-variant rescued cells increase the autophagic flux, whereby the increased supply of (mitochondrial nucleotide) waste products surpasses endogenous baseline levels. These could further burden already dysfunctional lysosomes packed with ssDNA species. For instance, and of relevance for AD pathogenesis, APP C-terminal fragments (APP-CTFs) have been shown to become partially degraded through the autophagy-lysosome route, whereas their aberrant accumulation also contributes to the autophagic and endolysosomal pathology observed in neuronal models of AD^11,12,64–66^. Given the similarities with our observations in PLD3-defective cells, we considered a possible contribution of altered APP proteolysis. Western blot analysis showed that APP-CTFs, the direct substrate of amyloid-β (Aβ) peptides, significantly accumulated in PLD3^−/−^ and most SNP variant-expressing cells compared to xWt rescued cells (Fig. 6h). Surprisingly, also levels of secreted Aβ peptides were elevated (Fig. 6i), arguing that a decreased lysosomal proteolysis by cathepsins (Fig. 4c) and/or a decreased access to γ-secretase in lysosomes^67^, rather than a lowered intramembrane proteolysis could explain APP-CTF accumulation. Because part of APP is also proteolyzed through the autophagic route^64,68^, we considered that the build-up of APP-CTFs could be linked to the cGAS-STING induced increased autophagic flux in PLD3^−/−^ and SNP-variant expressing cells.

Using an antibody (82E1) that recognizes the N-terminus of β-cleaved APP-CTF, we confirmed with confocal imaging the accumulation and increased co-localization of APP-CTF in LC3-positive autophagosomes/autolysosomes in PLD3^−/−^, xM6R and xV232M cells compared to Wt-rescued cells (Fig. 6d–g). A 5 h treatment with H151 significantly lowered APP-CTF levels, as shown by western blotting (Fig. 6j) and confocal imaging (Fig. 6d, f), as well as its co-localization with LC3-positive compartments (Fig. 6g). Prolonged exposure with H151 up to 24 h further decreased APP-CTF levels back to those of Wt-rescued cells (Fig. 6k, l). Inhibition of TBK1 also lowered APP-CTF accumulation, albeit not to the same extent (Fig. 6k, l). Interestingly, activating STING with cGAMP, the product of cGAS that binds to STING, caused an increase in APP-CTF levels and over a period of 24 h treatment (Fig. 6k, l). Overall, these data underscore that STING hyperactivation is a main contributor to APP-CTF accumulation observed in PLD3^−/−^ and SNP variant expressing cells.

### Knockout of APP alleviates autophagy-lysosome pathology in PLD3-defective cells

**Fig. 7:**
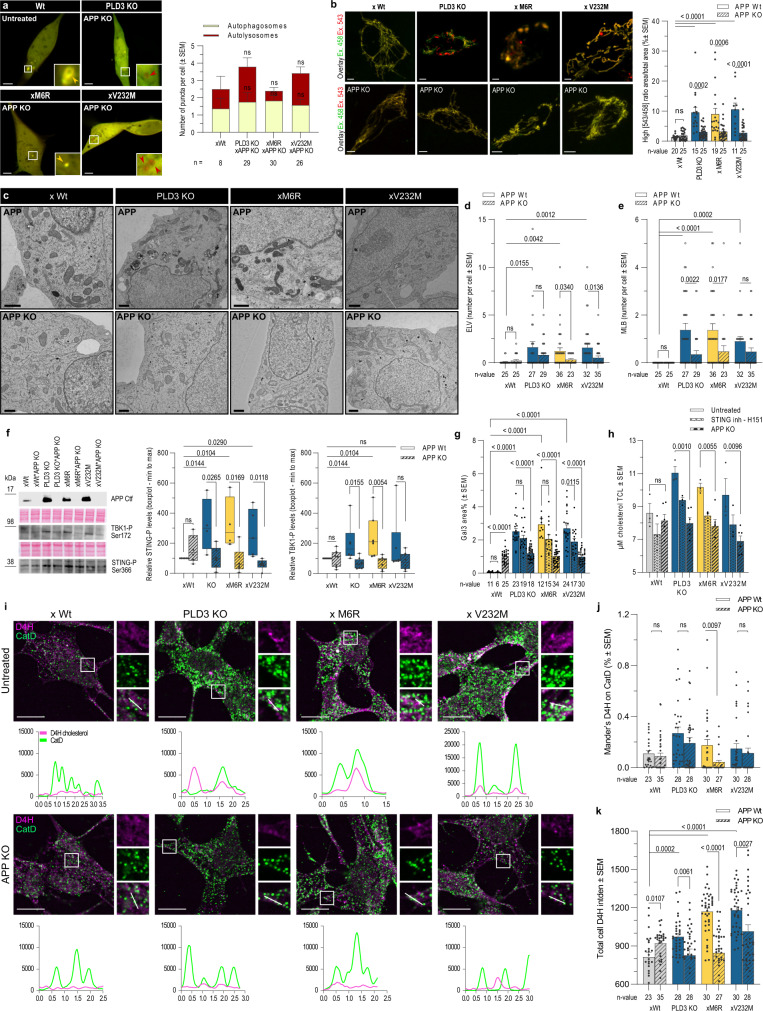
APP depletion and STING inhibition ameliorate endolysosomal pathology upon PLD3 dysfunction. **a** Representative airyscan images of autophagosome/-lysosome puncta as in Fig. 6b, using SH-SY5Y knocked out for APP. These show LC3 puncta levels indistinguishable from xWt levels. Scale bar = 5 µm. Statistical significance was calculated using two-tailed, unpaired *t*-tests (specific *n*-values are listed on the graph). **b** The index of mitophagy in APP KO cells was calculated using mt-Keima (scale bar = 5 µm). The graph shows a high [543/458] ratio area/total mitochondrial area. An ordinary one-way ANOVA with Bonferroni’s multiple comparisons test was used (*n* = 11–25). **c** Representative transmission electron microscopy (TEM) images showing a rescue effect of an APP KO in a PLD3 dysfunction background. Counts of **d** electron-lucent vesicles (ELV) and **e** multilamellar bodies (MLB) per cell as identified on TEM images (*n* = 25–36). Scale bar = 1 µm. Two-tailed, unpaired *t*-tests were used for statistical testing. **f** APP knockout double transgenics were analyzed on western blot for STING activation (*n* = 6) and TBK1 autophosphorylation at Ser172 (*n* = 8). Statistical differences were calculated using two-tailed, unpaired *t*-tests and depicted on boxplots (min to max; median as the center, the box extends from the 25th to 75th percentiles, and whiskers range from the smallest to the greatest value). **g** Quantification of Gal3 levels on confocal images (*n* = 6–34). Statistical significance was calculated using an ordinary one-way ANOVA with Bonferroni’s multiple comparisons test. **h** Beneficial effect on cholesterol levels in total cell lysates (TCL; *n* = 3 in untreated and H151 conditions, *n* = 6 in the APP KO conditions) after 5 h H151 treatment or after an APP KO, using the Cholesterol Glo Assay. Statistical differences were calculated using two-tailed, unpaired *t*-tests. **i** Top: representative confocal images of untreated and APP knockout SH-SY5Y cells, stained with the D4H-cholesterol probe (magenta) and CatD (green). Images were taken in confocal mode on a ZEISS LSM 900. Scale bar = 10 µm. Bottom: Co-localization analysis using line intensity profiles. Quantification of IF images using **j** Manders’ coefficient and **k** integrated densities per cellular area (*n* = 23–35). Statistical differences were calculated using two-tailed, unpaired *t*-tests as indicated. Source data are provided as a Source Data file.

Given APP-CTF contributes to endolysosomal dysfunction, we next considered a synergistic effect of APP-CTF accumulation. We, therefore, investigated whether a knock-out of APP would ameliorate the autophagic-lysosomal pathology in PLD3^−/−^, xM6R, and xV232M vs xWt cells. Irrespective of a PLD3^−/−^ or a SNP-variant background, APP deficiency clearly lowered the number of autophagosomes and autolysosomes to levels indistinguishable from the xWt condition (Fig. 7a). Concomitantly, we observed a restoration of the mitophagy process as demonstrated by the reduced mKeima-Red-Mito-7 levels in PLD3^−/−^, xM6R and xV232M rescued cells (Fig. 7b). This was also reflected in a significantly increased co-localization of Lysotracker with Mitotracker and an amelioration of lysosome-mitochondria MCSs, albeit not significant (Fig. S6a). The normalization of mitophagy and autophagy correlated as well with a significant decrease in ELVs and MLBs in APP-deficient PLD3^−/−^ and SNP variant rescued cells (Fig. 7c–e). Importantly, both pSTING and pTBK1 levels were as well strongly reduced (Fig. 7f). This might be explained by the lowered lysosomal leakiness (Figs. 7g and S6b, c), activating cGAS-STING. Collectively, these findings imply a feed-forward loop with APP-CTF accumulation not only being a downstream effect of STING hyperactivation in PLD3^−/−^ and SNP variant rescued cells, but also being an accelerating factor through aggravating autophagic-lysosomal pathology.

For example, alterations in cholesterol metabolism are a common feature in AD pathology and are at least partially contributed by APP proteolytic fragments^69^. In addition, APP silencing was shown to reduce cholesterol content and synthesis, attributed to a halving of nucleus-translocated sterol regulatory element binding protein 2 (SREBP2)^70^. An APP KO indeed reduced total cholesterol levels. Inhibiting STING also reduced total cholesterol levels, but to a minor extent compared to an APP^−/−^ (Fig. 7h). An APP^−/−^ indeed lowered the (autophago)lysosomal accumulation of the membrane cholesterol sensor, D4H* in our PLD3^−/−^, xM6R and V232M-rescued cells, while not affecting xWt rescued cells (Fig. 7i–k). Given that APP deficiency also reduced total cholesterol levels in these cells (Fig. 7h), we reasoned that de novo cholesterol biosynthesis might be affected. Indeed, the ratio of cleaved, activated SREBP2 over the inactive, uncleaved form was significantly elevated in PLD3^−/−^ and SNP-variant expressing cells (Fig. S6d–f). Inhibiting STING (Fig. S6d, e) as well as knocking out APP (Fig. S6d, f) reverted SREBP2 activation. In contrast, the low-density lipoprotein receptor-related protein 1 (LRP1), another lipid metabolism protein, was not affected by an altered PLD3 expression nor STING inhibition or APP^−/−^ (Fig. S6d), underscoring the specificity of the observed effects on cholesterol metabolism.

## Discussion

Our data support a critical role for nucleotide turnover in autophagy-lysosomal homeostasis. Herein we identify PLD3 as a key lysosomal 5’−3’ exonuclease with a major activity towards mtDNA, as evidenced by the enrichment of mitochondrial genes in lysosomes isolated from PLD3^−/−^ and PLD3 SNP variant rescued neuroblastoma cells. This mtDNA build-up originated from a decreased capacity of lysosomes to degrade dysfunctional mitochondria through PINK1-dependent mitophagy, causing a degradative bottleneck. The latter is supported as well by an overt lysosomal pathology, including a reduced catabolic activity, an increased propensity for leakage, the accumulation of ELVs and MLBs, and a marked increase in lysosomal cholesterol content. We further describe a mechanism whereby decreased membrane integrity may allow mtDNA to leak from lysosomes to activate cytosolic cGAS-STING signaling, autophagy, and disrupted APP catabolism. This is of potentially high interest regarding PLD3 being a LOAD risk gene^18,71^ and wherein an altered exonuclease activity may hyper-activate STING signaling, initiating crosstalk between mitochondrial dysfunction, APP, and cholesterol metabolism-all prominent cellular hallmarks in AD- converging here.

These cellular perturbations are observed in both PLD3^−/−^ and SNP-variant expressing cells. Surprisingly, this is not solely due to a loss-of-function of PLD3 as some SNPs, including M6R and T426A, show an increased exonuclease activity (Fig. 1i, j). Interestingly, although the xM6R rescued line displays lower lysosomal DNA concentrations, its electropherogram shows peaks not present in xWt-expressing cells. A possible explanation is that the M6R variant would shift substrate cleavage, generating less degradable DNA fragments. In line, a previous study showed the PLD3 exonuclease kinetics tend to be higher for substrates with a T 5’-base, followed by A, G, and C^29^. While we show altered PLD3 kinetics to have a clear impact on DNA metabolism, our data do not support a role for PLD3 as a critical (or essential) RNAse, as was suggested previously^31^. However, these authors only showed such activity in total extracted RNA samples, while not differentiating between nucleotide species in enriched lysosomes. Hence, it cannot be excluded that in vivo, PLD3 degradation of RNA is compensated by lysosomal RNAses, including RNase T2, that degrade exogenous RNA molecules^72^. Given PLD3 is highly abundant in neurons and neuronal-derived cell lines, including SH-SY5Y neuroblastoma^21^, and PLD4 is mainly expressed in immune cells, we can neither disregard that different expression profiles of PLD3 vs PLD4 could underlie tissue and/or cell-specific differences in RNA metabolism.

In addition, we demonstrate a clear preference for PLD3 activity towards CpG-rich DNA fragments. This favored CpG-specificity is maintained by SNP variants, with the exception of the N236S variant that shifts its preference to CpG-poor substrates (Fig. 1b). Of note, a too excessive build-up of CpG-rich DNA fragments can be detrimental to the brain. Mice receiving chronic intracerebroventricular infusions with CpG-DNA show marked axonal damage, impaired neural function, and ensuing neurodegeneration^73,74^. In an immune context, PLD3/4 degrades microbial DNA, which is enriched in unmethylated CpG motifs^30,42^. Looking at a more neuronal context, mtDNA resembles microbial DNA with respect to CpG-content. Indeed, our study shows that PLD3 dysfunction correlates with mtDNA accretion in lysosomes; identifying mtDNA as a major substrate for lysosomal PLD3 in neuron-like cells. These findings correlate strongly with the robust mitophagy increases in PLD3^−/−^ and SNP-variant expressing cells. Alongside, ultrastructurally, MLBs accumulate, often with mitochondrial remnants, likely caused by a deficit in the lysosomal degradative capacity. The failure to properly degrade mtDNA leads to a build-up of fragments, triggering topologically distinct nucleotide-based signaling pathways. Our results in SH-SY5Y cells show that a defective PLD3 function increases TLR9 levels and downstream target genes. While intuitively, one would associate over-activity with pathology, TLR9 activity has been ascribed with both beneficial and detrimental effects. Microglial TLR9 activation elicits and sustains a Th1-dominated immunopathogenic reaction^75^. On the other hand, patients with amnestic mild cognitive impairment progress less likely to clinical AD when they show high TLR9 levels^76^. In addition, the TLR9 p.E317D mutation, situated in the TLR9 substrate pocket, reduces its activity by half and segregates in AD families^77^.

Our finding that cytosolic cGAS-STING signaling is also activated in PLD3^−/−^ and SNP-variant expressing cells not only strengthens its potential in AD neuropathogenesis^78^, but, more importantly, molecularly links this pathway for the first time to a LOAD risk gene; PLD3. Whereas our data suggest that this activation results from a lysosomal leakage of mtDNA upon defective mitophagic removal, its direct release from (dysfunctional) mitochondria is also possible^79,80^, or both. Given the essential role of PLD3 as a lysosomal exonuclease, our data also underscore that lysosomal defects may well be initiated upstream of mitochondrial dysfunction in LOAD. In support, a lysosome-centered cascade has been described upon different genetic and pharmacological interferences with lysosomal functioning, including in different neuropathologies. Parkinson’s disease-associated mutations in ATP13A2, GBA, or VPS13C all cause lysosome-driven mitochondrial defects^56,81–84^, as do FTLD-linked mutations in progranulin^85^. Such propagation of defects may have multiple causes. We show an altered PLD3 activity to affect the lysosomal Ca^2+^ storage and to reduce MCS duration and length between lysosomes and mitochondria. These MCSs are described to regulate key mitochondrial Ca^2+^ dynamics as well as mitochondrial fission^53,54,86^. In addition, an altered PLD3 function significantly impacts the lysosomal lipidome, which could also negatively impact a normal exchange of metabolites between lysosomes and mitochondria. Although part of the lipid changes could originate from the decreased capacity of PLD3-defective autolysosomes to degrade membranes, as evidenced by the accretion of MLBs, specific changes may be more directly impacting mitochondrial metabolism; such as decreased levels of PE and specific MGs. The latter exceed their role as energy storage molecules and have been associated with functions in mitochondrial metabolism, oxidative stress, and cellular senescence^49^. The PE metabolism is vital for mitochondria functionality and is imported into mitochondria through MCSs^87^. Our results also underscore a significant accumulation of lysosomal cholesterol, which may as well affect lysosome-mitochondria cholesterol import and, as such, mitochondrial membrane fluidity^52^. Once distorted, repercussions have been shown on, i.a., mitochondrial glutathione import and on ATP generation^88,89^. In accordance, we detected affected mitochondrial bioenergetics in PLD3 models.

In this study, mtDNA, delivered to lysosomes through mitophagy, is a major and constant source of PLD3 substrates in neuronal cells. When PLD3’s exonuclease activity is defective, lysosomes become dysfunctional, debris starts to build up within, and there is an increased propensity to lysosomal leakage and, as such, STING pathway activation. Lysosomal membrane damage and associated leakage was also shown to induce STING activation in a model of Niemann-Pick type C disease^57,90^. Where STING signaling leads to a similar inflammatory response as does TLR9 activation, both pathways have been linked to the autophagy process as well^57,59^. Our data indeed show increased TBK1 phosphorylation and high mito/autophagic levels that get significantly reduced upon STING or TBK1 inhibition and, to a lesser extent, TLR9 inhibition.

In agreement with other studies and PLD3 being a LOAD risk gene^22,33,41^, altering PLD3 activity significantly impacted APP metabolism. In addition to increased Aβ secretion, we found a concomitant accumulation of the direct precursor, APP-CTF, which we found unexpectedly to be STING activation-dependent. Whereas this build-up could result from a lowered access to γ-secretase, alternatively, it is known that part of APP escapes the canonical dual processing and is degraded through autophagy/lysosomes^68,91,92^. The reduced degradative capacity and accretion of autolysosomes in PLD3^−/−^ and SNP-variant-expressing cells could explain the build-up of APP-CTFs, as evidenced by its localization in LC3- and CatD-positive autolysosomes (Figs. 6d, g and S5b). Several independent studies in iPSC-derived human neurons expressing familial AD-associated mutations in APP and PSEN1 demonstrated a causal connection between APP-CTF accumulation and endolysosomal abnormalities^11,12,65,66^. Our study now extends this similar, if not identical, APP-CTF toxicity to a LOAD risk gene, PLD3. Surprisingly, APP deficiency also lowered STING activation, suggesting an additional layer of crosstalk between STING pathway activation, autophagy induction, and APP-CTF accumulation in PLD3^−/−^ and SNP variant-expressing cells. Moreover, APP-CTF activated SREBP2, causally linking APP-CTF cytotoxicity to de novo cholesterol synthesis and lysosomal cholesterol handling, providing an additional feed-forward loop that promotes lysosomal dysfunction in the context of altered PLD3 expression.

A dysfunctional lysosomal compartment likely also impacts endosomal transport and recycling; for one causing cholesterol redistribution^93^. For instance, clathrin-mediated APP endocytosis is modulated by cholesterol levels^94^. The increased cholesterol levels we observe in PLD3-defective cells may, hence, impact APP internalization and endosomal routing, promoting its amyloidogenic processing as observed here (Fig. 6i). A PLD3 knockdown was demonstrated to reduce the number of SNX1-decorated tubules, indicating as well crosstalk with endosomal recycling through the retromer^22^. Reduced retromer-dependent recycling may lead to an endosomal traffic jam, as shown in studies of the retromer receptor SORL1^95^. While SORL1 itself is becoming upgraded from a risk gene to likely a causal gene in AD, several other LOAD risk genes impinge on endolysosomal transport regulation, rendering it a causal pathway in LOAD pathogenesis^1,4,18,71^. To what extent functionally connected risk genes indeed co-occur in individuals and contribute to the polygenic risk of developing LOAD is an exciting and challenging topic for future research.

**Fig. 8:**
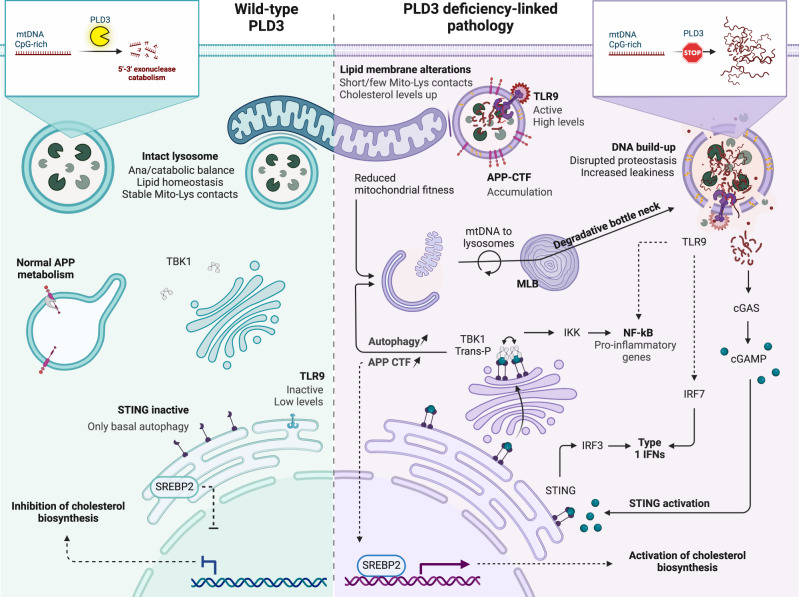
PLD3 exonuclease dysfunction impacts CpG-rich mtDNA catabolism that promotes a more broadly lysosome-centered pathology. PLD3 depletion and exonuclease dysfunction-causing SNPs promote lysosomal mtDNA build-up. This is accompanied by a lysosomal catabolic impairment and an increased leakiness propensity, leading to TLR9 and STING pathway activation that further promotes autophagy. This results in the accumulation of APP-CTF in autolysosomes, providing additional crosstalk with STING activation, while upregulating SREBP2, and activating de novo cholesterol synthesis. Overall, we identify several feed-forward loops that further confound the degradative capacity of lysosomes, resulting in the accumulation of autolysosomes. A decreased lysosomal Ca^2+^ storage/release and a significantly altered lipid composition likely impact as well mitochondria-lysosome MCSs, further propagating the lysosome-centered defects to other organelles, notably mitochondria. Created with BioRender.com.

Overall, an attractive picture is emerging wherein the failure of PLD3-deficient lysosomes to degrade mtDNA instigates nucleotide signaling pathways, including TLR9 and cGAS-STING. Both activate downstream autophagy, but the failure of lysosomes to keep pace with degradation results in the particular accumulation of autolysosomes, surpassing the share of autophagosomes. Herein, our results identify several feed-forward loops propelling lysosomal dyshomeostasis (Fig. 8). The build-up of undegraded material may compromise lysosomal integrity further, adding to the cytosolic mtDNA levels that uphold STING activation. The parallel build-up of APP-CTFs in autolysosomes induces de novo cholesterol synthesis and alters cholesterol handling, compromising normal lysosomal lipid composition and lysosomal Ca^2+,^^96^. This may impact the communication and exchange through MCSs, spreading the lysosome-centered pathology to other organelles, including mitochondria, and causing mitochondrial fitness to drop even more.

The significant impact of PLD3 on APP metabolites, as we demonstrate here, emphasizes the importance of studying PLD3 in a neuronal context. As the defective nucleotide signaling pathways connect PLD3 not only to autophagy but also to inflammation, this predicts that similar mechanisms could impact other AD-relevant cell types, in particular microglia. Although almost absent under homeostatic conditions, PLD3 expression gets systematically upregulated in the activated response microglia (ARMs) state. Independent transgene and APP knock-in models show an up to fourfold increase in PLD3 in ARMs^97–99^. A major focus for future studies could be to investigate to what extent PLD3 SNPs contribute to the exacerbated inflammatory state that is characteristic of AD progression and to what extent activated nucleotide signaling pathways, as shown in this study, are involved.

## Methods

### Antibodies

The PLD3 C-terminal antibody was generated by immunization of rabbits with the human PLD3 C-terminal peptide WDSPYSHDLDTSADSVGNAC (Eurogentec). The peptide was fused to keyhole limpet hemocyanin using the Imject Maleimide Activated Carrier Protein Spin Kit (Thermo Scientific), according to the manufacturer’s instructions. New Zealand white rabbits were boosted four times at 1-month time intervals (P191/2017, Ethics Committee KU Leuven). The resulting sera were peptide-affinity purified on NHS Sepharose fast flow 75% slurry (Cytiva) and concentrated with Vivaspin 15 R centrifugal tubes (25 min spin at 3000 × *g*, VWR). The resulting purified antibody was aliquoted with an equal amount of glycerol and stored at −20 °C until use.

Other antibodies used in this paper include: rabbit anti-APP (Y188, Ab32136, Abcam, 1:10.000 for WB, 1:500 for IF), mouse anti-APP-CTF (82E1, IBL Co., 1:100 for IF), rabbit anti-ATG5 (2630, Cell Signaling Technology, 1:500 for WB), rabbit anti-Cathepsin-D (ab75852, Abcam, 1:1000 for WB, 1:100 for IF), rat anti CD289-Y585 (TLR9-PE, eB72-1665, eBioscience, 1:200 for FC), mouse anti-cytochrome C (556433, BD Biosciences, 1:1000 for WB), rat anti-galectin-3 (M3/38, Sc-23938, Santa Cruz, 1:100 for IF), mouse anti-GAPDH (MAB374, EMD Millipore, 1:10.000 for WB), mouse anti-IFN-α-R670 (APC, 130-099-214, Miltenyi Biotec, 1:10 for FC), mouse anti-LAMP1 (611042, BD Biosciences, 1:500 for WB), rat anti-Lamp1 (1D4B sc19992, Santa Cruz, 1:50 for IF), mouse anti-LAMP1 (CD107a, H4A3, BioLegend, 1:200 for IF), rabbit anti-LC3 B (NB600-1384, Novus Biologicals, 1:1000 for WB, 1:100 for IF), rabbit anti-Lrp1 (ab92544, Abcam, 1:1000 for WB), mouse anti-GM130 (610823, BD Biosciences, 1:50 for IF), mouse anti-Na+K+ ATPase alpha (#NB300-146, Novus Biologicals, 1:2000 for WB), rabbit anti-PINK1 (BC100-494, Novus Biologicals, 1:1000 for WB), rabbit anti-N-terminal PLD3 (in-house generated^29^, 1:1000 for WB), mouse anti-Rab7 (ab50533, Abcam, 1:500 for WB), rabbit anti-SREBP2 (10007663, Cayman Chemical, 1:1000 for WB), rabbit anti-STING (D1V5L, 50494 S, Cell signaling, 1:500 for WB, 1:50 for IF), rabbit anti-STING-P at Ser366 (D7C3S, 19781 S, Cell signaling, 1:500 for WB), rabbit anti-TBK1/NAK (D1B4, 3504 S, Cell signaling, 1:500 for WB), rabbit anti-TBK1/NAK at Ser172 (D52C2, 5483 S, Cell signaling, 1:500 for WB), rabbit anti-TLR9 (PA5-20203, Thermo Fisher Scientific, 1:250 for IF), mouse anti-TNF-α-VS25 (BV510, 502949, Biolegend, 1:10 for FC), and goat anti-VPS35 (Ab10099, Abcam, 1:250 for IF).

### Compounds

Following drugs were used as indicated in the corresponding sections: H151 (STING-inhibitor, 1 µM, 5 h for WB/IF, Invivogen), tlrl-bx7 (TBK1/IKKε inhibitor, 1 µM, 2-4-6-24 h for WB, 5 h for IF, Invivogen), cGAMP (STING-ligand/activator, 2 µg/mL, 2-4-6-24-48 h for WB, 5 h for IF, Invivogen), TLR-BW006 Class B CpG oligonucleotide (TLR9-ligand/activator, 2.5 µM, 2-4-6-24-48 h for WB, 5 h for IF, Invivogen), ODN-INH-18 Class B inhibitory (TLR9-inhibitor, 2.5 µM, 2-4-6-24-48 h for WB, 5 h for IF, Invivogen), and bafilomycin (vascular H^+^-ATPase inhibitor, 200 nM, 6 h for IF, BioConnect).

### Generation of stable cell lines

#### Knockout cell lines

The Alt-R CRISPR-Cas9 technology from IDTDNA was used for the generation of *PLD3* and *APP* knockout cell lines in the SH-SY5Y (ATCC, CRL-2266) background; authentication was performed by DNA-STR-Typing at the Leibniz-Institut on 19.02.2016 (No.: A1602030-1). Two independent PLD3^−/−^ lines were generated in which either Ex5 (TACTCGCAAGGGTCATAGCA) or Ex6 (AGTAGAAGGAGGCGATGTCC) was targeted by the sgRNA. The *APP* gene was targeted by a combination of two sgRNAs, recognizing sequences in Ex3 (ACAGTGGAGGCTTGTTAGATGC and GAGTCCAAAACACAGTACAACAC). In each condition, 700,000 cells were electroporated with 30 pmol of sgRNA complex and 10 pmol/µL of Alt-R® S.p. Cas9 Nuclease V3 (Intergrated DNA Technologies) suspended in R-buffer of the Neon Transfection System (Thermo Fisher). Pulse conditions consisted of three pulses of 1200 V and a 20 ms pulse width. Knockout pools were subcloned and clones of interest were validated with the c-terminal PLD3 Ab on western blot and sequenced through the Eurofins Sequencing Service (Belgium). Genome edits were subsequently assessed by sequence trace decomposition, using the TIDE software^100^.

#### Rescue cell lines

cDNA construct generation was previously described in ref. ^29^. These sequences were now cloned towards a pLJC5 vector backbone (Lentiviral vector type), using Gibson assembly. For this, PLD3 sequences were first amplified with the Q5 High-Fidelity 2X Master Mix (NEB) and the following primers: ccgtttttggcttttttgttagacgaagcgctagcatgaagcctaaactgatgtaccaggagctgaagg and atgaatactgccatttgtctcgaggtcgagaattctcagagcaggcggcagg. Gibson assembly was carried out with the NEBuilder HiFi DNA Assembly Cloning Kit (NEB), according to the manufacturer’s instructions. After transformation in Stbl3 cells (Thermo Fisher Scientific), constructs were sequenced with the following primers: CGAGTGTGTTTTGTGAAG and CTACTATTCTTTCCCCTGC. For lentiviral production, HEK293T (CRL-3216, ATCC) were co-transfected with the plasmid of interest, a packaging construct (pCMVR8.74) and a VSV-G envelope expressing plasmid (pMD2.G), using FuGENE6 (Promega) according to the manufacturer’s protocol. Particle-containing mediums was collected 24 h after transfection and filtered through a 0.45 µm filters (PALL 4184, VWR). Serial dilutions were made of the viral particles that were added to the cells in a medium containing polybrene (1:1000, H9268, Sigma). After 24 h, puromycin selection was initiated (3 µg/ml, P8833, Sigma). Stable pools were validated by western blot analysis.

#### PINK1 knockdown

Cells were transfected with ON-TARGETplus human PINK1 siRNA—SMARTpool (L-004030-00-0005; Horizon Discovery) at a concentration of 20 uM siRNA and using jetPRIME transfection reagent (Westburg). Cells were analysed 24 h after transfection.

### Transmission electron microscopy

SH-SY5Y cells were grown in 10 cm culture dishes up to 90–95% confluence and, subsequently, fixed in 2.5% glutaraldehyde (Agar Scientific), sodium cacodylate buffer (0.1 M, pH 7.2) at 4 °C (overnight). Fixed cells were washed thrice (10 min) to remove glutaraldehyde, using sodium cacodylate buffer. The cells were scraped in sodium cacodylate buffer and centrifuged at 200×*g* at room temperature (RT). The resulting pellets were resuspended in 1.5% agarose (in 0.1 M sodium cacodylate buffer), centrifuged at 400×*g* at RT, and solidified on ice for 30 min. The embedded pellets were post-fixed with 1% osmium tetroxide, and 1.5% ferrocyanide in a 0.1 M cacodylate buffer for 2 h. After rinsing with dH_2_O, the pellets were dehydrated in a graded ethanol series (30, 50, 70%). Each incubation was performed on a rotor at 4 °C for 10 min. Samples were stained en bloc with 4% uranyl acetate in 70% ethanol for 30 min at 4 °C in the dark. Further dehydration steps were carried out in 90 and 100% ethanol. Following dehydration, samples were infiltrated with epoxy resin/propylene oxide mixtures (50 and 66%). The next day, cell pellets were embedded with 100% epoxy resin in inverted BEEM-capsules (Agar Scientific, 2 days, 60 °C). Ultrathin sections of 70 nm were cut on an Ultracut S Ultramicrotome (Leica Reichert) and post-stained with 4% uranyl acetate in water (10 min) and Reynolds’ lead citrate (5 min)^101,102^. Micrographs were taken at 80 KV on a JEM-1400 transmission electron microscope (Jeol) that is equipped with a Quemesa 11 Mpxl camera (Olympus). The distance of EL-mitochondrion contacts was measured with the RADIUS 2.0 (Build 14402, Emsis) software.

### mCherry-D4H* probe

The pGEX-KG-D4H*-mCherry plasmid (Addgene # 134604; ref. ^103^) was transformed (50 ng) in DH5-alpha Competent *E. coli* cells by a heat shock (42 °C, 30 s) and induced by 0,4 mM isopropyl-β-d-1-thiogalactopyranoside (IPTG). Incubation was carried out for 20 h (18 °C, 180 rpm). The resulting cell pellet was resuspended (buffer A+, containing 50 mM lysozyme, 2 mM DTT, and protease inhibitor cocktail (PIC) without EDTA; 11836170001, Roche), sonicated (1 min pulse − 1 min rest program, times 4 on a Branson Sonifier 250) and the obtained lysed cells cleared from debris (30 min, 5887×*g* on a TLA 110 rotor at 4 °C on Optima MAX Ultracentrifuge (Beckman Coulter)). The supernatant was loaded on Glutathion-B4-beads that were washed with PBS with PIC, removing nonspecific interactions. After a thrice-repeated incubation of the D4H*-probe (15 min at 4 °C) on an end-to-end rotor, the probe was eluted (50 mM tris buffer with 40 mM glutathione, 1x PIC, pH 8,0). The eluates were concentrated and washed with PBS-PIC in a 50 K MWCO filter in an Allegra X-15R Centrifuge (30 min, 4000×*g* at 4 °C). The probe was stored with 20 mV% sucrose at −20 °C until further use.

### Confocal microscopy

#### Immunofluorescence staining of coverslips

Cells were fixed in 4% PFA (10 min at RT), washed with PBS^+/+^, and semi-permeabilized in 0,1% Triton X-100 for 10 min (or using a 20 s dip in liquid nitrogen in case of D4H*-staining). A 1-h blocking step was performed (PBS + 2% BSA + 5% donkey serum, RT) to reduce background staining. Additionally, for cholesterol, coverslips were incubated with the GST-D4H*-mCherry probe (0,1 µg/µL, at RT for 2 h), re-fixed in PFA 4% for 10 min at RT, and washed thrice with PBS^+/+^. Next, primary antibodies, diluted in blocking buffer, were applied for 2 h at RT. After washing the coverslips thrice with PBS^+/+^, secondary antibodies were applied, and dissolved in blocking buffer as were the primary for 60 min at RT. The coverslips were mounted in Mowiol (Sigma) mounting solution, which was left to dry at RT (ON) before the slides were put at 4 °C for longer storage. Slices were imaged in confocal mode on a ZEISS LSM 900 and all image analysis was done using Image J software. To measure the area of the organelle of interest, segmentation was realized using automatic thresholding. Overlap of signals of interest were quantified with Manders co-localization index (Plugin Jacob).

#### Live-imaging mKeima

The mt-Keima construct (mt/mKeima/pIND(SP1)) was a gift from Dr. A. Miyawaki (RIKEN Brain Science Institute, Japan)^104^. Cells were transfected using electroporation (Neon Transfection System, Thermo Fisher Scientific) in a 10 μL volume containing 700,000 cells and 2 μg DNA. The device was set at three pulses of 1200 V (pulse voltage) and 20 ms (pulse width). After 24 h, cells were imaged on a Leica TCS SP5 II confocal microscope equipped with a 63x objective lens (HC PL APO 63x/1.4 CS2), a multi-argon laser (458, 476, 488 nm) and a He/Ne laser (543 nm). Mt-Keima was imaged in two channels via two sequential excitations (458 nm, green; 543 nm, red) and using a 600 to 695 nm emission range. Image analysis was performed with the Image J software, using the Ratio Plus plugin as previously described^44^. The index of mitophagy was calculated as the parameter: high [543/458] ratio area/total mitochondrial area.

#### Mitochondria-lysosome co-localization analysis

Cells were incubated with MitoTracker Deep Red (50 nM, M22426, Fisher) and LysoTracker Green DND-26 (50 nm, L7526, Fisher). For image acquisition, an inverted Zeiss LSM 880 with a fast airyscan microscope was used in combination with a 60x Plan Apochromat (1.40) oil objective. Images (*n* = 500) were taken at an interval of 0.25 s and definite focus applied. The setup was controlled by ZEN black (version 2.3, Carl Zeiss Microscopy GmbH). For analysis, an Image J custom macro was used to normalize both channels, using CLIJX/CLI2, and to detect the thresholded lysosome (channel 1, red, min area [px] = 1) and mitochondria (channel 2, green, min area [px] = 20) binary masks. The colocalization area was measured by time point and normalized to the total amount of lysosomes. For contact duration measurements, lysosomes were tracked using PALM-Tracer (max distance 8, min length 15). From the result script, the intervals were assessed during which co-localization was present at a minimum contact area of 1 px (threshold method: Moments; spot diameter: 10).

#### PicoGreen localization analysis

The fluorescent DNA dye PicoGreen was used to label (nuclear and) mtDNA within living cells. To this end, Quant-iT PicoGreen dsDNA Reagent (P7581, Thermo Fischer) was added at 3 μl/ml to the cell culture medium and incubated for 1 h (37 °C). Mitotracker (10 min incubation) and Lysotracker (no extra incubation, added straight to HBSS imaging medium) were used at a concentration of 50 nM. High-speed live confocal imaging was performed on a ZEISS LSM 880 in fast Airyscan mode. Co-localization was analysed with the Jacob plugin (Fiji).

#### pH and autophagic flux analysis

Cells were transfected with 1 µg plasmid per 100 µl of Opti-MEM medium, containing 0.5% FuGENE HD Reagent (Promega). pFUGW-FIRE-pHLy was obtained from Addgene (plasmid # 170774)^105^ and the mCherry-GFP-LC3 was in house available^106^. High-speed live confocal imaging was performed on a ZEISS LSM 880 in fast Airyscan mode. For pH analysis, we calculated the raw integrated density (RawIntDen) values of the lysosomal ROI in Fiji. The mTFP1/mCherry signal ratio was plotted as a measurement of lysosomal pH. For autophagic flux assessment, vesicles with both mCherry (red) and GFP (green) were counted as autophagosomes, while those with only a mCherry signal were counted as autolysosomes.

#### Lysosomal Ca^2+^ responses

Calcium responses were measured essentially as previously described in ref. ^107^. Briefly, on the day of recording, cells were loaded with 1 µM Fura-2 AM solution (F1221, Thermo Fisher Scientific) for 45 min at RT. Cells were rinsed twice with Krebs-Ca^2+^ (135 mM NaCl, 5.9 mM KCl, 11.5 mM Glucose, 11.6 mM HEPES, 1.2 mM MgCl_2_, and 1.5 mM CaCl_2_) and left at RT for 30 min for de-esterization. All acquisitions were carried out in Krebs-EGTA^+^ (135 mM NaCl, 5.9 mM KCl, 11.5 mM Glucose, 11.6 mM HEPES, 1.2 mM MgCl_2_, and 2 mM EGTA). Cells were pulsed with 20 µM of TRPML activator ML-SA1 (Cat. No. 4746, Tocris) and the response was acquired every 5 s for 10 min, using an Olympus IX81 with a UAPO/340 40x oil objective (1.35 NA) and operated by CellR Software (Olympus). Recordings were done at 340 and 380 nm excitation (530 nm emission filters). For image analysis, cells were segmented manually in Image J and fluorescence intensities were measured over time. Fura-2 signals were corrected to obtain ΔF/F0 (F0 being the initial signal recorded). In addition, each cell’s area under the curve was measured using Prism GraphPad 9.2.0.

### Flow cytometry

All flow cytometric analyses were performed on a BD Fortessa X-20 analyzer and the stopping gate was set at 10.000 events. Data were analyzed using FlowJo (v10; Treestar, Ashland, OR, USA). The FSC-A/SSC-A plot was used to eliminate debris and the FSC-H/FSC-A dot plot for identifying singlets.

#### MitoProbe JC1 assay

1E06 cells were incubated with 2 µM JC1 mitochondrial membrane potential probe (T3168, Thermo Fisher Scientific) for 30 min at 37 °C (5% CO_2_) before analysis. JC1 responsiveness to membrane potential alterations was validated with carbonyl cyanide 3-chlorophenylhydrazone (CCCP, C2759, Sigma-Aldrich)-mediated mitochondrial depolarization (6 h, 30 µM). Cell populations were plotted in contour plots and the percentage of mitochondrial depolarization was indicated.

#### Intracellular staining

Protein secretion was inhibited in 1E06 cells by incubation with monensin (2 µM, 00-4505-51, Invitrogen) for 3 h at 37 °C. Intracellular Staining was performed following the Foxp3 Fixation/Permeabilization buffer set protocol (00-5523-00, eBioscience) and listed antibodies. mean fluorescent intensity (MFI) values were calculated using FlowJo.

#### Lysosomal permeability

1E06 cells were stained with 50 nm of lysosomotropic dye (LysoTracker Red DND-99, L7528, Thermo Fisher Scientific) and LIVE/DEAD™ Fixable Green Dead Cell Stain (L34969, Thermo Fischer Scientific) for 15 min. Positive control conditions were pre-incubated with 1 mM LLOMe (L7393, Sigma) for 10 min at 37 °C and STING inhibition conditions with 1 μM H151 (941987-60-6, Sanbio) for 5 h. The MFI was calculated and the leakiness propensity was calculated as the +LLOME/untreated ratio. For Gal3 staining, see the confocal microscopy section.

#### Lysosomal function according to the DQ-Red BSA lysosomal protease activity assay (D12051, Invitrogen)

Cells were incubated with 10 µg/ml DQ-Red BSA and 50 nM LysoTracker green for 30 min at 37 °C. After two wash steps, cells were analyzed at four time points (0, 30, 60, and 90 min), allowing for kinetic slope calculation. Activities were analyzed relative to the LysoTracker signal.

### Mitochondrial bioenergetic measurements

The Seahorse XF Cell Mito Stress Test Kit (103015-100, Agilent Technologies) was used to measure the cells’ oxygen consumption rate (OCR) and extracellular acidification rate (ECAR). ATP production, maximal respiration, and non-mitochondrial respiration were calculated from the OCR after the addition of 1 µM oligomycin, 1 µM FCCP, and a mix of 0,5 µM rotenone and antimycin A, respectively, using the Agilent/Seahorse XF Report Generator software. Experiments were performed on 65.000 cells/well in a complete Seahorse XF DMEM medium (103680-100, Agilent Technologies).

### End-labeled fluorescence-quenched oligonucleotide (EFQO) assay

The EFQO assay was performed as previously described in ref. ^29^. Briefly, the assay uses fluorophore- and fluorescence-quencher-labeled oligonucleotides to assess the 5’ exonuclease activity at a pH of 5 (MES buffer). Used sequences with varying degrees of CpG-content include random reference sequence (/56-FAM/accatgatgttcctgatgctaagtatg*c*a*c*/3IABkFQ/, /56-FAM/accatgacgttcctgatgctaagtatg*c*a*c*/3IABkFQ/ and /56-FAM/accatgacgttcctgacgctaagtacg*c*a*c*/3IABkFQ/), the MT-ATP6 gene (/56-FAM/agccctggctgtatgcctaactgctaa*c*a*t*/3IABkFQ/, /56-FAM/agccctggccgtacgcctaaccgctaa*c*a*t*/3IABkFQ/ and /56-FAM/agccctggccgtatgcctaactgctaa*c*a*t*/3IABkFQ/) and the MT-ND4L gene (/56-FAM/agtctttgctgcctgtgaagcagtggt*g*g*g*/3IABkFQ/, /56-FAM/agtctttgccgcctgcgaagcagcggt*g*g*g*/3IABkFQ/, and /56-FAM/agtctttgctgcctgtgaagcagcggt*g*g*g*/3IABkFQ/). The * indicates a PTO bond, excluding 3’−5’ exonuclease activity. As 5’-Mod we used 6-Fam (qPCR probe) and at the 3’ end the Iowa Black FQ. Oligos were ordered from IDT. Fluorescent signals were acquired for a 12 h time-frame with 5 min intervals on a GloMax Multi Detection Plate Reader (Promega).

### Isolation of late endosomes/lysosomes using superparamagnetic iron oxide nanoparticles (SPIONs)

The production and use of SPIONs for high-yield purification of late endosomes and lysosomes, compatible with (omics) analysis, can be found as a step-by-step guide in ref. ^108^.

### Real-time polymerase chain reaction (qPCR)

Total DNA was extracted from cytosolic fractions (supernatant resulting from ultracentrifuging the PNS; 417,200 × *g*, 30 min) and endolysosomal isolates from SH-SY5Y cells, using 15 µg of protein isolates on the Nucleospin DNA RapidLyse (MN 740100.250, filterservice). qPCR was conducted on a LightCycler® 480 Real-Time PCR System (Roche). Each reaction contained extracted DNA (1 µl), along with LightCycler® 480 SYBR® Green I Master (REF 04707516001, Roche) and 150 nM of each primer: rTBP (F- CGGCTGTTTAACTTCGCTTC, R-CACACGCCAAGAAACAGTGA), rUBC (F-ATTTGGGTCGCGGTTCTTG, R-TGCCTTGACATTCTCGATGGT), dTBP (F-GGCATCTGTCTTTGCACACC, R-GGGTCAGTCCAGTGCCATAA), dUBC (F-TGGCACAGCTAGTTCCGTC, R-TCGCGGACCCAGACTACAG), mt-ATP6 (F-AATCCAAGCCTACGTTTTCACA, R-AGTATGAGGAGCGTTATGGAGT), mt-CO2 (F-AATCGAGTAGTACTCCCGATTG, R-TTCTAGGACGATGGGCATGAAA), mt-D-LOOP (F-CTATCACCCTATTAACCACTCA, R-TTCGCCTGTAATATTGAACGTA), and mt-ND1 (F-CACCCAAGAACAGGGTTTGT, R-TGGCCATGGGTATGTTGTTAA). Samples were run (60 cycles) in technical duplicates and gene copies were calculated relatively to 100,000 copies of hTBP, as previously described in ref. ^109^.

### Lysosomal nucleotide content

Qubit 4 Fluorometer. Fluorescence-based quantification was used to detect ssDNA (Q10212), dsDNA (Q33230), and RNA (Q32852) in PNS and isolated lysosomes, according to the manufacturer’s instructions (Thermo Fischer Scientific). Protein levels (Q33211) were measured for normalization purposes.

Sizing and quantification on the Agilent 2100 Bioanalyzer System. DNA 1000 Lab Chips (5067-1504, Agilent Technologies) were loaded with samples as recommended by the manufacturer. After gel preparation, each sample well was loaded with 1 μl of lysosome extract or PNS and with 5 μl of the internal marker. The marker mixture for the DNA 1000 Lab Chip contains lower and upper molecular size markers of 15 and 1500 bp, respectively. Electropherograms, virtual gel images, and table data were analyzed using Agilent 2100 Expert Software (Agilent Technologies).

### Lipidomics

Lipid analysis was performed at Lipometrix—KU Leuven Lipidomics Core Facility (Leuven, Belgium). An amount of sample containing 10 ug of protein was diluted in 700 μl water and mixed with 800 μl 1 N HCl:CH3OH 1:8 (v/v), 900 μl CHCl3, 200 μg/ml of the antioxidant 2,6-di-tert-butyl-4-methylphenol (BHT; Sigma-Aldrich), 3 μl of SPLASH® LIPIDOMIX® Mass Spec Standard (#330707, Avanti Polar Lipids), and 3 μl of Ceramides and 3 μl of Hexosylceramides Internal Standards (#5040167 and #5040398, AB SCIEX). After vortexing and centrifugation, the lower organic fraction was collected and evaporated using a Savant Speedvac spd111v (Thermo Fisher Scientific) at room temperature and the remaining lipid pellet was stored at −20 °C under argon.

Just before mass spectrometry analysis, lipid pellets were reconstituted in 100% ethanol. Lipid species were analyzed by liquid chromatography-electrospray ionization tandem mass spectrometry (LC-ESI/MS/MS) on a Nexera X2 UHPLC system (Shimadzu) coupled with hybrid triple quadrupole/linear ion trap mass spectrometer (6500 + QTRAP system; AB SCIEX). Chromatographic separation was performed on an XBridge amide column (150 mm × 4.6 mm, 3.5 μm; Waters) maintained at 35 °C using mobile phase A [1 mM ammonium acetate in water-acetonitrile 5:95 (v/v)] and mobile phase B [1 mM ammonium acetate in water-acetonitrile 50:50 (v/v)] in the following gradient: (0–6 min: 0% B → 6% B; 6–10 min: 6% B → 25% B; 10–11 min: 25% B → 98% B; 11–13 min: 98% B → 100% B; 13–19 min: 100% B; 19–24 min: 0% B) at a flow rate of 0.7 mL/min, which was increased to 1.5 mL/min from 13 min onwards. SM, CE, CER, and HCER were measured in positive ion mode with a precursor scan of 184.1, 369.4, 264.4, and 264.4, respectively. TAG, DAG, and MAG were measured in positive ion mode with a neutral loss scan for one of the fatty acyl moieties. PC, LPC, PE, LPE, PG, PI, and PS were measured in negative ion mode by fatty acyl fragment ions. Lipid quantification was performed by scheduled multiple reactions monitoring (MRM), the transitions being based on the neutral losses or the typical product ions as described above. The instrument parameters were as follows: Curtain gas = 35 psi; Collision gas = 8 a.u. (medium); IonSpray voltage = 5500 and −4,500 V; Temperature = 550 °C; Ion source gas 1 = 50 psi; Ion source gas 2 = 60 psi; Declustering potential = 60 and −80 V; Entrance potential = 10 and −10 V; Collision cell exit potential = 15 and −15 V.

The following fatty acyl moieties were taken into account for the lipidomic analysis: 14:0, 14:1, 16:0, 16:1, 16:2, 18:0, 18:1, 18:2, 18:3, 20:0, 20:1, 20:2, 20:3, 20:4, 20:5, 22:0, 22:1, 22:2, 22:4, 22:5, and 22:6 except for TGs which considered: 16:0, 16:1, 18:0, 18:1, 18:2, 18:3, 20:3, 20:4, 20:5, 22:2, 22:3, 22:4, 22:5, and 22:6. Peak integration was performed with the MultiQuant^TM^ software version 3.0.3. Lipid species signals were corrected for isotopic contributions (calculated with Python Molmass 2019.1.1) and were quantified based on internal standard signals and adheres to the guidelines of the Lipidomics Standards Initiative (LSI) (level 2 type quantification as defined by the LSI). GraphPad Prism software 9.2.0 (San Diego, CA, USA) was used for statistical calculations and graphing.

### Aβ peptide quantification in a conditioned medium by MSD-ELISA

The MesoScale Discovery (MSD) multispot Aβ ELISA was performed as previously described in ref. ^110^. Briefly, 72-h-conditioned medium of SH-SY5Y cells was mixed 1:1 in blocking buffer (PBS supplemented with 0.1% casein), of which 50 μl/well was loaded on a multi-Spot 96-well MSD-ELISA plate. Capture antibodies were JRF/cAb40/28 for Aβ40, JRF/cAb42/26 for Aβ42, and the detection antibody was JRF/AbN/25 raised against the N-terminus of Aβ. After overnight incubation and 5 wash steps, 150 μl/well of the 2x MSD Read Buffer T (Tris-based buffer containing tripropylamine) was added to develop signals that were read on a Sector Imager 6000 (Meso Scale Discovery). Concentrations were calculated from standards (synthetic human Aβ1–40 and Aβ1–42 peptides) at known concentrations.

### Cellular cholesterol measurement

Total cellular cholesterol levels were measured using the Cholesterol Glo™ Assay (J3190, Promega, USA) according to the manufacturer’s description. A total of 5000 cells was used and the H151 (1 µM) condition was incubated for 1 h with the compound before lysis.

### Statistical analysis

All graphs and statistical analyses were generated using GraphPad Prism v9.2.0 (Prism). Both the type of post-hoc test as well as the p-values are indicated in the figure legends. For all experiments, data were reported based on individual cells or biological replicates pooled from multiple cells, e.g., lysates. No statistical methods were used to predetermine the sample size.

### Reporting summary

Further information on research design is available in the Nature Portfolio Reporting Summary linked to this article.

## Supplementary information

Supplementary Information

Description of Additional Supplementary Files

Supplementary Movie 1

Supplementary Movie 2

Supplementary Movie 3

Supplementary Movie 4

Supplementary Data 1

Reporting Summary

## Data Availability

All data were available in the main text or the supplementary materials. Raw lipidomic data, as well as an Excel with all calculated values, have been made available at the NIH Common Fund’s National Metabolomics Data Repository (NMDR) website, the Metabolomics Workbench [https://www.metabolomicsworkbench.org], where it has been assigned Project ID PR001497 [10.21228/M8740F]. Source data are provided with this paper.

## Source data

Source Data
