# Supplementary material for: Phospholipase D3 degrades mitochondrial DNA to regulate nucleotide signaling and APP metabolism
Source: Nat Commun. 2023 May 24;14:2847. doi: 10.1038/s41467-023-38501-w (PMC10209153; doi:10.1038/s41467-023-38501-w)
Supplement: Supplementary file 2 — Description of Additional Supplementary Files [file 41467_2023_38501_MOESM2_ESM.pdf]

Title: Supplementary Movie 1.
Description: Fast airyscan microscopy imaging of mitochondria-lysosome contacts in xWt SH-SY5Y cells, stained with MitoTracker Deep Red and LysoTracker Green. Images (n = 500) were taken at an interval of 0.25s and movies generated at 7 frames/seconds. The scale bar = 1 µm. Videos support data of figure 4.

Title: Supplementary Movie 2.
Description: Fast airyscan microscopy imaging of mitochondria-lysosome contacts in PLD3 KO SH-SY5Y cells, stained with MitoTracker Deep Red and LysoTracker Green. Images (n = 500) were taken at an interval of 0.25s and movies generated at 7 frames/seconds. The scale bar = 1 µm. Videos support data of figure 4.

Title: Supplementary Movie 3.
Description: Fast airyscan microscopy imaging of mitochondria-lysosome contacts in xM6R SH-SY5Y cells, stained with MitoTracker Deep Red and LysoTracker Green. Images (n = 500) were taken at an interval of 0.25s and movies generated at 7 frames/seconds. The scale bar = 1 µm. Videos support data of figure 4.

Title: Supplementary Movie 4.
Description: Fast airyscan microscopy imaging of mitochondria-lysosome contacts in xV232M SH-SY5Y cells, stained with MitoTracker Deep Red and LysoTracker Green. Images (n = 500) were taken at an interval of 0.25s and movies generated at 7 frames/seconds. The scale bar = 1 µm. Videos support data of figure 4.

Title: Supplementary data 1
Description: Html file containing graphical representations of all lipid classes analyzed, with the option for both a quantitative and relative quantitation display.
