# Supplementary material for: Phospholipase D3 degrades mitochondrial DNA to regulate nucleotide signaling and APP metabolism
Source: Nat Commun. 2023 May 24;14:2847. doi: 10.1038/s41467-023-38501-w (PMC10209153; doi:10.1038/s41467-023-38501-w)
Supplement: Supplementary file 1 — Supplementary Information [file 41467_2023_38501_MOESM1_ESM.pdf]

Supplementary Materials for

PLD3 degrades mitochondrial DNA to regulate nucleotide signaling and APP metabolism

Zoë P. Van Acker, Anika Perdok, Ruben Hellemans, Katherine North, Inge Vorsters, Cedric Cappel, Jonas Dehairs, Johannes V. Swinnen, Ragna Sannerud, Marine Bretou, Markus Damme, Wim Annaert
Correspondence to: wim.annaert@kuleuven.be

This PDF file includes:

Figure legends for S1 to S6
Captions for Movies S1 to S4

Other Supplementary Materials for this manuscript include the following:

Movies S1 to S4
Supplementary data - Lipidomics

Fig. S1. SH-SY5Y PLD3 model systems. (a) PLD3 protein structure with indicated SNPs in red. (b) TEM of internalized SPIONs in SH-SY5Y cells: given their small size (10 nm), SPIONS are readily targeted to endosomes/lysosomes (EL) following a 16h chase without interference with their normal functions or endosomal routing. Scale bar = 0.5 µm. ER = endoplasmic reticulum, Mit. = mitochondrion. (c) TEM image of isolated SPION-loaded EL, essentially devoid of contaminants as routinely checked according to (Tharkeshwar *et al.*, PMID: 33377016). Scale bar = 0.5 µm. (d) Representative WB result of a SPION-isolated fraction (mean ± SEM, n = 3). The blot has been probed for organelle markers. TCL - total cell lysate; Bnd - Bound/SPION-isolated fraction. (e) WB on 15 µg of TCL and 5 µg of SPION-purified endolysosomal extracts (EL), using our new PLD3 C-terminal pab (1:5000) and the previously described N-terminal Ab. (1:1000). The PLD3 luminal domain accumulates in EL (55 kDa). Cross-reactivity is observed with mouse PLD3, though SH-SH5Y cells have the highest endogenous levels. The antibody gives reproducible results and has been checked on samples of 2 independent PLD3 KO SH-SY5Y clones for specificity. (f) Endogenous c-terminal fragment levels of PLD3 in TCL, using our new C-terminal pab (1:5000). Given their neuronal origin and accordingly high PLD3 levels, SH-SY5Y neuroblastoma cells were chosen for the generation of PLD3 knockout cells that were validated at (g) the transcript levels with qPCR analysis, using a primer pair at either of the independently used cutting sites (n = 3), and (h) the protein level. Different concentrations of lentiviral vectors were used for generating PLD3 rescue lines, continuing with those rescues with endogenous-like PLD3 levels. (i) WB on 6 µg of PNS and 2 µg of isolated EL. The cleavage ratio of PLD3 was calculated as the c-terminal fragment:full-length ratio. The C-terminal pab was used at 1:5000. Source data are provided as a Source Data file.

Fig. S2. PLD3 alterations affect the lysosomal system and mitochondrial fitness. (a) JC1 MitoProbe assay. JC1 aggregates accumulated inside healthy mitochondria in a membrane-potential dependent manner. (b-d) 1E06 cells were incubated with 2 µM JC1 for 30 minutes at 37°C (5% CO2) and analysed on a BD Fortessa X-20 analyser to detect the JC1 monomers and aggregates. The stopping gate was set at 10.000 events. (b) Gating strategy. (d) JC1 responsiveness to membrane potential alterations was validated with CCCP-mediated mitochondrial depolarization (6h, 30 µM). (e) Representative TEM images of PLD3 SH-SY5Y cell variants. Two independent sample preps from 2 PLD3 clonal lines were analysed per genotype. Knockouts and SNP-carrying cells show clear electron-lucent vesicles (ELV), i.e. immature multivesicular bodies (MVB) that accumulate at the crossroad of recycling and degradation, as well as multilamellar bodies (MLB). Scale bar is 1 µm. (f) Probing of the lysosomal pH in our SH-SY5Y model, using the genetically encoded, pH-Sensitive mTFP1 Biosensor, showing no significant pH changes except with bafilomycin (6 hours 200 nM). TLR9 activation (Class B CpG oligonucleotide, 2.5 µM, 5h) and STING activation (mammalian (non-canonical) CDN, cyclic [G(2',5')pA(3',5')p], 2 µg/ml, 5h) were validated alongside an APP knockout. An ordinary one-way ANOVA with Bonferroni's multiple comparisons test was used for statistical testing (n = 23-38). Representative images (left); Scale bar = 5 µm. Source data are provided as a Source Data file.

Fig. S3. PLD3 dysregulations impact the endolysosomal (membrane/lipid) compartment. (a) Western blot analysis of lysosomal markers (n = 6). Two-tailed, unpaired t tests were used for statistical analyses. (b) Lipidomics data, with lipid species being quantified by one deuterated standard per lipid class. Sum by unsaturations (% of a whole) for either the endolysosomal (EL) fraction and postnuclear supernatant (PNS). (c) Similarly, the sum by carbon (% of a whole) is depicted. Features were calculated from individual quantities of lipid molecular species (mean, n=3). (d) Pie chart with functional lipid class subclassifications: (i) glycerophospholipids (GPLs): phosphatidylcholine/-ethanolamine/-inositol/-serine/-glycerol (PC/PE/PI/PS/PG) and acyl-linked PC/PE (-O, -P), and Diacylglycerides (DG), (ii) sphingolipids (SP): ceramide (Cer), hexosylceramide (HexCer) and sphingomylein (SM), (iii) storage: Triacylglycerides (TG) and cholesterol esters (CE) and (IV) others: Lysophosphatidylcholine (LPC), Monoacylglycerides (MG), Lysophosphatidylethanolamine (LPE). Values are standardized to nmol | mg protein within the sample (n = 3). (e) Lipidomics data as in panel d, depicted per lipid class. Mean values of biologically independent samples are represented in the bar graphs. (f) Quantitative cholesterol levels (n = 3). (g) Volcano plots show log2 fold-changes versus −log10 P-values of lysosomal lipid species compared to the Wt-rescue value. T-tests for −log10 P-values were two-tailed. Each plot has different classes highlighted for visibility. Source data are provided as a Source Data file.

Fig. S4. TLR9 gets activated upon a dysregulated lysosomal ssDNA catabolism linked to PLD3 dysregulations. (a) Intracellular flow cytometry of inflammatory components. Mean Fluorescence Intensity (MFI) triplicate measurements were made with the stopping gate set at 10000 events. An ordinary one-way ANOVA with Bonferroni's multiple comparisons test was used for statistical testing. Differences with the wild-type rescue condition are indicated. (b) Up: representative confocal images of PLD3 SH-SY5Y models stained for TLR9 (red) and LAMP1 (green) in confocal-mode on a ZEISS LSM 900. Scale bar = 10 µm. Down: Colocalization analysis using line intensity profiles. (c/d) Quantification of b images using (c) area volume and (d) Manders' coefficient (n = 21, 24, 28 and 25 cells for the xWt, KO, xM6R and xV232M condition, respectively). Statistical differences were calculated using two-tailed, unpaired t-tests as indicated. (e) Gating strategy for the increased lysotracker positive area (Fig. 4a) and DQ-BSA assay (Fig. 4c). (f) Gating strategy for the increased vulnerability to LLOMe-induced leakiness (Fig. 5c). Source data are provided as a Source Data file.

Fig. S5. cGAS-STING activation links PLD3 dysfunction with APP CTF and cholesterol build-up. (a) Graph shows the beneficial effect on cholesterol levels in total cell lysates (TCL, n = 3) after 5h H151 treatment, using the Cholesterol Glo Assay. Statistical differences were calculated, using two-tailed, unpaired t-tests. (b) Top: representative confocal images of untreated and H151-treated SH-SY5Y cells, stained with the D4H-cholesterol probe (magenta), CatD (green) and APP CTF (82E1 antibody, blue). Images were taken in confocal-mode on a ZEISS LSM 900. Scale bar = 10 µm. Bottom: Co-localization analysis using line

intensity profiles. (c/d) Quantification of IF images using Manders' coefficient and area volume (n = 21-38). Statistical differences were calculated using two-tailed, unpaired t-tests as indicated. (e) Left: Representative airyscan images of autophagosome (mCherry+GFP positive) and autolysosome puncta (mCherry positive), using mCherry-GFP-LC3 in untreated or PINK1 KD SH-SY5Y cells. Scale bar = 5 µm. Right: Quantification of Airscan images. Statistical differences were calculated with two-tailed, unpaired t-tests in between autolysosome (indicated in red) and autophagosome (indicated in green) numbers (specific n-values are listed on the graph). Source data are provided as a Source Data file.

Fig. S6. APP depletion ameliorates autophagy-lysosome pathology in cells with an affected PLD3 function. (a) Bar graphs showing the mean percentage overlap between lysosomes and mitochondria as well as their contact duration, measured from airyscan time-lapse images. APP double transgenics' values are striped. Two-tailed, unpaired t-tests were used for statistical testing (individual contacts analysed in n = 10, 13, 15 and 10 cells – xWt*APP KO, PLD3 KO*APP KO, xM6R*APP KO and xV232M*APP KO cells – over t = 125 s). (b) Up: representative confocal images of PLD3 SH-SY5Y models stained for CatD (green) and Gal3 (magenta) in confocal-mode on a ZEISS LSM 900. Scale bar = 10 µm. Down: Colocalization analysis using line intensity profiles. (c) Beneficial effect of H151-induced STING inhibition and an APP KO on lysosomal leakiness propensity. Scale bar = 10 µm. (d) SREBP2 has a ~130 kDa, inactive full-length form as well as ~60 kDa, active N-terminal fragment. Representative blot of independent experiments on STING inhibition and an APP KO, quantified in e (n = 5) and f (n = 4), respectively. Two-tailed, unpaired t-tests were used for statistical testing. Source data are provided as a Source Data file.

Supplementary video legends

Movie S1.

Fast airyscan microscopy imaging of mitochondria-lysosome contacts in xWt SH-SY5Y cells, stained with MitoTracker Deep Red and LysoTracker Green. Images (n = 500) were taken at an interval of 0.25s and movies generated at 7 frames/seconds. The scale bar = 1 µm. Videos support data of figure 4.

Movie S2.

Fast airyscan microscopy imaging of mitochondria-lysosome contacts in PLD3 KO SH-SY5Y cells, stained with MitoTracker Deep Red and LysoTracker Green. Images (n = 500) were taken at an interval of 0.25s and movies generated at 7 frames/seconds. The scale bar = 1 µm. Videos support data of figure 4.

Movie S3.

Fast airyscan microscopy imaging of mitochondria-lysosome contacts in xM6R SH-SY5Y cells, stained with MitoTracker Deep Red and LysoTracker Green. Images (n = 500) were taken at an interval of 0.25s and movies generated at 7 frames/seconds. The scale bar = 1 µm. Videos support data of figure 4.

Movie S4.

Fast airyscan microscopy imaging of mitochondria-lysosome contacts in xV232M SH-SY5Y cells, stained with MitoTracker Deep Red and LysoTracker Green. Images (n = 500) were taken at an interval of 0.25s and movies generated at 7 frames/seconds. The scale bar = 1 µm. Videos support data of figure 4.

Supplementary data – Lipidomics

Excel file containing data from all lipid classes analyzed, with tabs on quantitative and relative quantitation values.

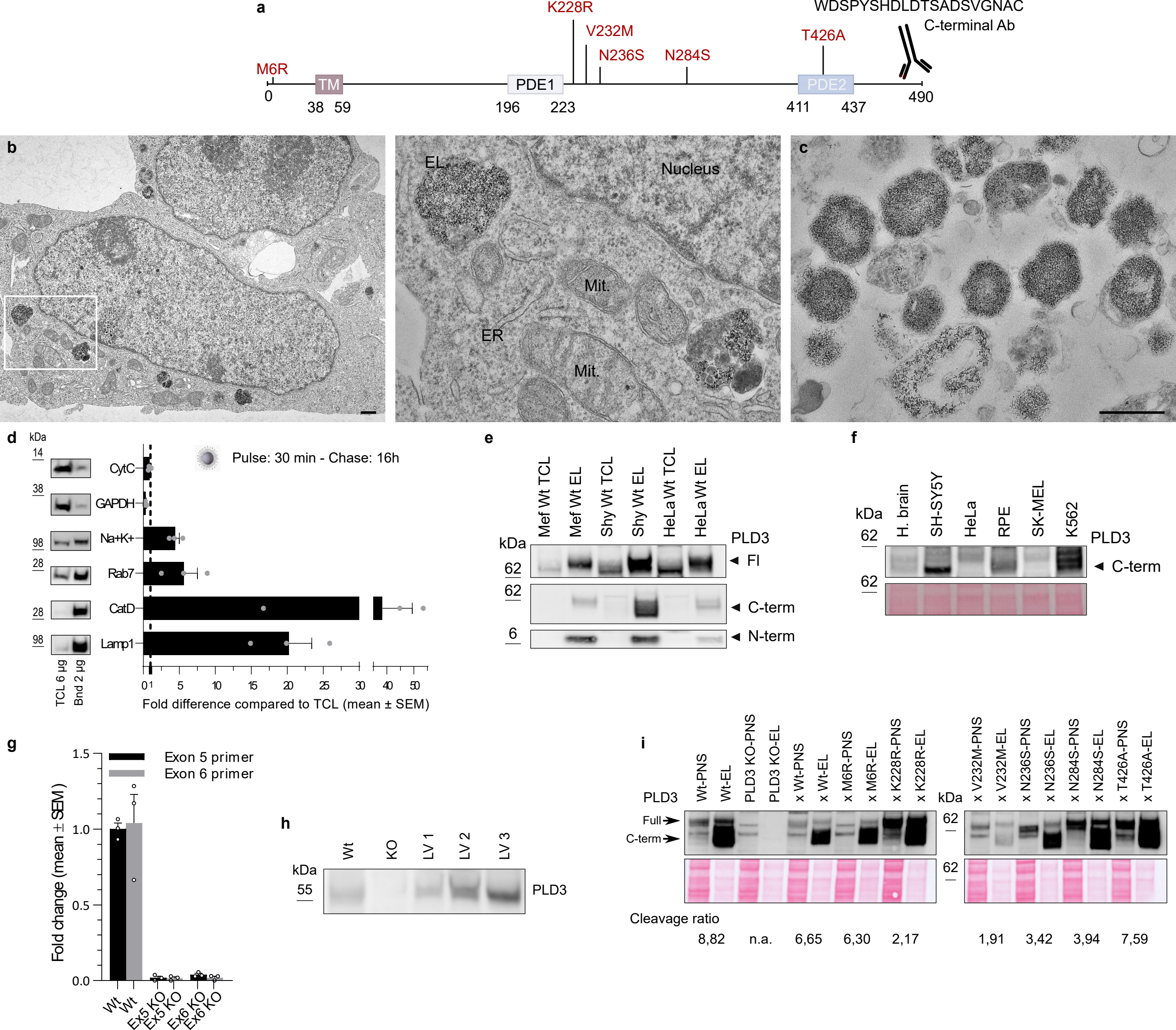
a
WDSPYSHDLDTSADSVGNAC
C-terminal Ab
M6R
K228R
V232M
N236S
N284S
T426A
TM
PDE1
PDE2
0
38 59
196 223
411 437
490
b
EL
Nucleus
Mit.
ER
Mit.
c
d
kDa
14
38
98
28
28
98
CytC
GAPDH
Na+K+
Rab7
CatD
Lamp1
TCL 6 µg
Bnd 2 µg
Pulse: 30 min - Chase: 16h
0 1 5 10 15 20 25 30 40 50
Fold difference compared to TCL (mean ± SEM)
e
Mef Wt TCL
Mef Wt EL
Shy Wt TCL
Shy Wt EL
HeLa Wt TCL
HeLa Wt EL
PLD3
kDa
62
62
6
Fl
C-term
N-term
f
kDa
62
62
H. brain
SH-SY5Y
HeLa
RPE
SK-MEL
K562
PLD3
C-term
g
Exon 5 primer
Exon 6 primer
Fold change (mean ± SEM)
0.0
0.5
1.0
1.5
Wt
Wt
Ex5 KO
Ex5 KO
Ex6 KO
Ex6 KO
h
Wt
KO
LV 1
LV 2
LV 3
kDa
55
PLD3
i
PLD3
Wt-PNS
Wt-EL
PLD3 KO-PNS
PLD3 KO-EL
x Wt-PNS
x Wt-EL
x M6R-PNS
x M6R-EL
x K228R-PNS
x K228R-EL
kDa
x V232M-PNS
x V232M-EL
x N236S-PNS
x N236S-EL
x N284S-PNS
x N284S-EL
x T426A-PNS
x T426A-EL
Full
C-term
62
62
Cleavage ratio
8,82
n.a.
6,65
6,30
2,17
1,91
3,42
3,94
7,59

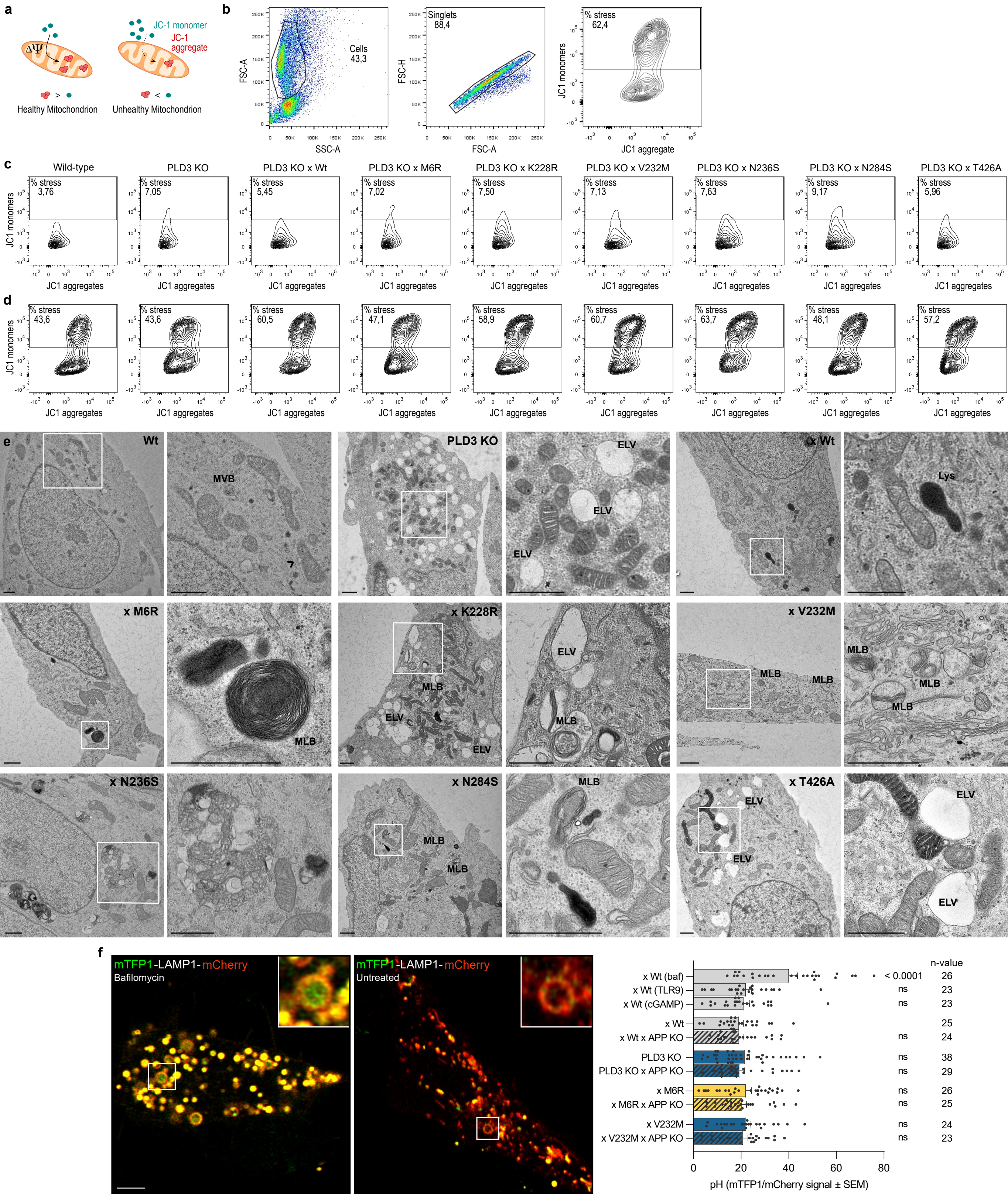
a
ΔΨ
JC-1 monomer
JC-1 aggregate
Healthy Mitochondrion
Unhealthy Mitochondrion
b
FSC-A
SSC-A
Cells 43,3
FSC-H
Singlets 88,4
FSC-A
% stress 62,4
JC1 monomers
JC1 aggregate
c
Wild-type
PLD3 KO
PLD3 KO x Wt
PLD3 KO x M6R
PLD3 KO x K228R
PLD3 KO x V232M
PLD3 KO x N236S
PLD3 KO x N284S
PLD3 KO x T426A
% stress 3,76
% stress 7,05
% stress 5,45
% stress 7,02
% stress 7,50
% stress 7,13
% stress 7,63
% stress 9,17
% stress 5,96
JC1 monomers
JC1 aggregates
d
% stress 43,6
% stress 43,6
% stress 60,5
% stress 47,1
% stress 58,9
% stress 60,7
% stress 63,7
% stress 48,1
% stress 57,2
JC1 monomers
JC1 aggregates
e
Wt
MVB
PLD3 KO
ELV
x Wt
Lys
x M6R
MLB
x K228R
MLB
ELV
x V232M
MLB
x N236S
x N284S
MLB
x T426A
ELV
f
mTFP1-LAMP1-mCherry
Bafilomycin
mTFP1-LAMP1-mCherry
Untreated
n-value
x Wt (baf) < 0.0001 26
x Wt (TLR9) ns 23
x Wt (cGAMP) ns 23
x Wt 25
x Wt x APP KO ns 24
PLD3 KO ns 38
PLD3 KO x APP KO ns 29
x M6R ns 26
x M6R x APP KO ns 25
x V232M ns 24
x V232M x APP KO ns 23
0 20 40 60 80
pH (mTFP1/mCherry signal ± SEM)

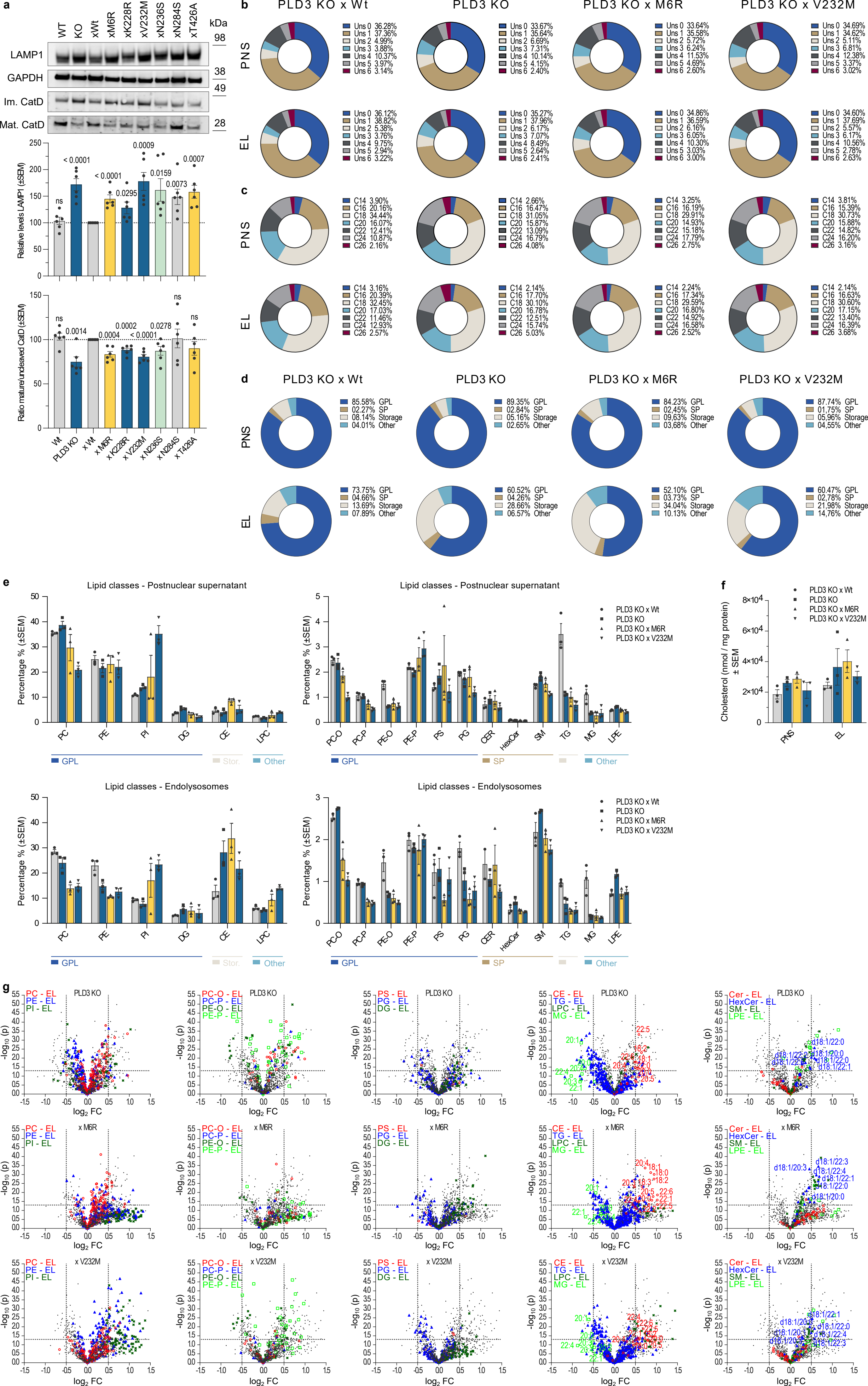
a
WT KO xWt xM6R xK228R xV232M xN236S xN284S xT426A
kDa
LAMP1
98
GAPDH
38
49
Im. CatD
Mat. CatD
28
Relative levels LAMP1 (±SEM)
ns
< 0.0001
< 0.0001
0.0295
0.0009
0.0159
0.0073
0.0007
Ratio mature/uncleaved CatD (±SEM)
ns
0.0014
0.0004
0.0002
< 0.0001
0.0278
ns
ns
Wt
PLD3 KO
x Wt
x M6R
x K228R
x V232M
x N236S
x N284S
x T426A
b
PLD3 KO x Wt
PLD3 KO
PLD3 KO x M6R
PLD3 KO x V232M
PNS
EL
Uns 0 36.28% Uns 1 37.36% Uns 2 4.99% Uns 3 3.88% Uns 4 10.37% Uns 5 3.97% Uns 6 3.14%
Uns 0 33.67% Uns 1 35.64% Uns 2 6.69% Uns 3 7.31% Uns 4 10.14% Uns 5 4.15% Uns 6 2.40%
Uns 0 33.64% Uns 1 35.58% Uns 2 5.72% Uns 3 6.24% Uns 4 11.53% Uns 5 4.69% Uns 6 2.60%
Uns 0 34.69% Uns 1 34.62% Uns 2 5.11% Uns 3 6.81% Uns 4 12.38% Uns 5 3.37% Uns 6 3.02%
Uns 0 36.12% Uns 1 38.82% Uns 2 5.38% Uns 3 3.76% Uns 4 9.75% Uns 5 2.94% Uns 6 3.22%
Uns 0 35.27% Uns 1 37.96% Uns 2 6.17% Uns 3 7.07% Uns 4 8.49% Uns 5 2.64% Uns 6 2.41%
Uns 0 34.86% Uns 1 36.59% Uns 2 6.16% Uns 3 6.05% Uns 4 10.30% Uns 5 3.03% Uns 6 3.00%
Uns 0 34.60% Uns 1 37.69% Uns 2 5.57% Uns 3 6.17% Uns 4 10.56% Uns 5 2.78% Uns 6 2.63%
c
PNS
EL
C14 3.90% C16 20.16% C18 34.44% C20 16.07% C22 12.41% C24 10.87% C26 2.16%
C14 2.66% C16 16.47% C18 31.05% C20 15.87% C22 13.09% C24 16.79% C26 4.08%
C14 3.25% C16 16.19% C18 29.91% C20 14.93% C22 15.18% C24 17.79% C26 2.75%
C14 3.81% C16 15.39% C18 30.73% C20 15.88% C22 14.82% C24 16.20% C26 3.16%
C14 3.16% C16 20.39% C18 32.45% C20 17.03% C22 11.46% C24 12.93% C26 2.57%
C14 2.14% C16 17.70% C18 30.10% C20 16.78% C22 12.51% C24 15.74% C26 5.03%
C14 2.24% C16 17.34% C18 29.59% C20 16.80% C22 14.92% C24 16.58% C26 2.52%
C14 2.14% C16 16.63% C18 30.60% C20 17.15% C22 13.40% C24 16.39% C26 3.68%
d
PLD3 KO x Wt
PLD3 KO
PLD3 KO x M6R
PLD3 KO x V232M
PNS
EL
85.58% GPL 02.27% SP 08.14% Storage 04.01% Other
89.35% GPL 02.84% SP 05.16% Storage 02.65% Other
84.23% GPL 02,45% SP 09,63% Storage 03,68% Other
87.74% GPL 01,75% SP 05,96% Storage 04,55% Other
73.75% GPL 04.66% SP 13.69% Storage 07.89% Other
60.52% GPL 04.26% SP 28.66% Storage 06.57% Other
52.10% GPL 03.73% SP 34.04% Storage 10.13% Other
60.47% GPL 02,78% SP 21,98% Storage 14,76% Other
e
Lipid classes - Postnuclear supernatant
Lipid classes - Endolysosomes
Percentage % (±SEM)
PC PE PI DG CE LPC
PC-O PC-P PE-O PE-P PS PG CER HexCer SM TG MG LPE
GPL Stor. Other SP
PLD3 KO x Wt
PLD3 KO
PLD3 KO x M6R
PLD3 KO x V232M
f
Cholesterol (nmol / mg protein) ± SEM
PNS
EL
g
PLD3 KO
x M6R
x V232M
PC - EL PE - EL PI - EL
PC-O - EL PC-P - EL PE-O - EL PE-P - EL
PS - EL PG - EL DG - EL
CE - EL TG - EL LPC - EL MG - EL
Cer - EL HexCer - EL SM - EL LPE - EL
-log₁₀ (p)
log₂ FC

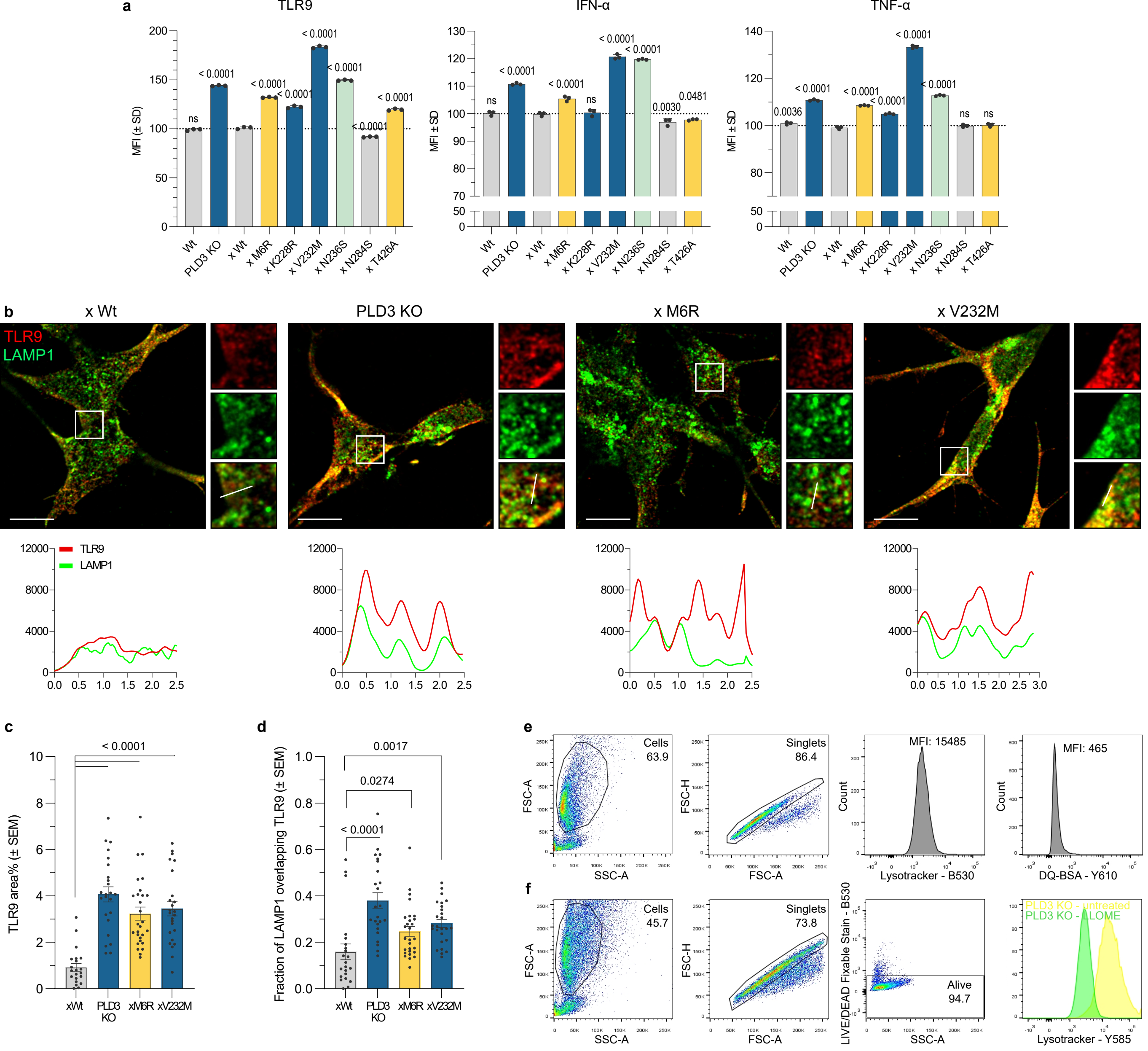
a
TLR9
IFN-α
TNF-α
MFI (± SD)
MFI ± SD
Wt
PLD3 KO
x Wt
x M6R
x K228R
x V232M
x N236S
x N284S
x T426A
ns
< 0.0001
0.0030
0.0481
0.0036
b
x Wt
PLD3 KO
x M6R
x V232M
TLR9
LAMP1
c
TLR9 area% (± SEM)
xWt
PLD3 KO
xM6R
xV232M
d
Fraction of LAMP1 overlapping TLR9 (± SEM)
0.0017
0.0274
e
Cells 63.9
Singlets 86.4
MFI: 15485
MFI: 465
FSC-A
SSC-A
FSC-H
Count
Lysotracker - B530
DQ-BSA - Y610
f
Cells 45.7
Singlets 73.8
Alive 94.7
LIVE/DEAD Fixable Stain - B530
PLD3 KO - untreated
PLD3 KO - LLOME
Lysotracker - Y585

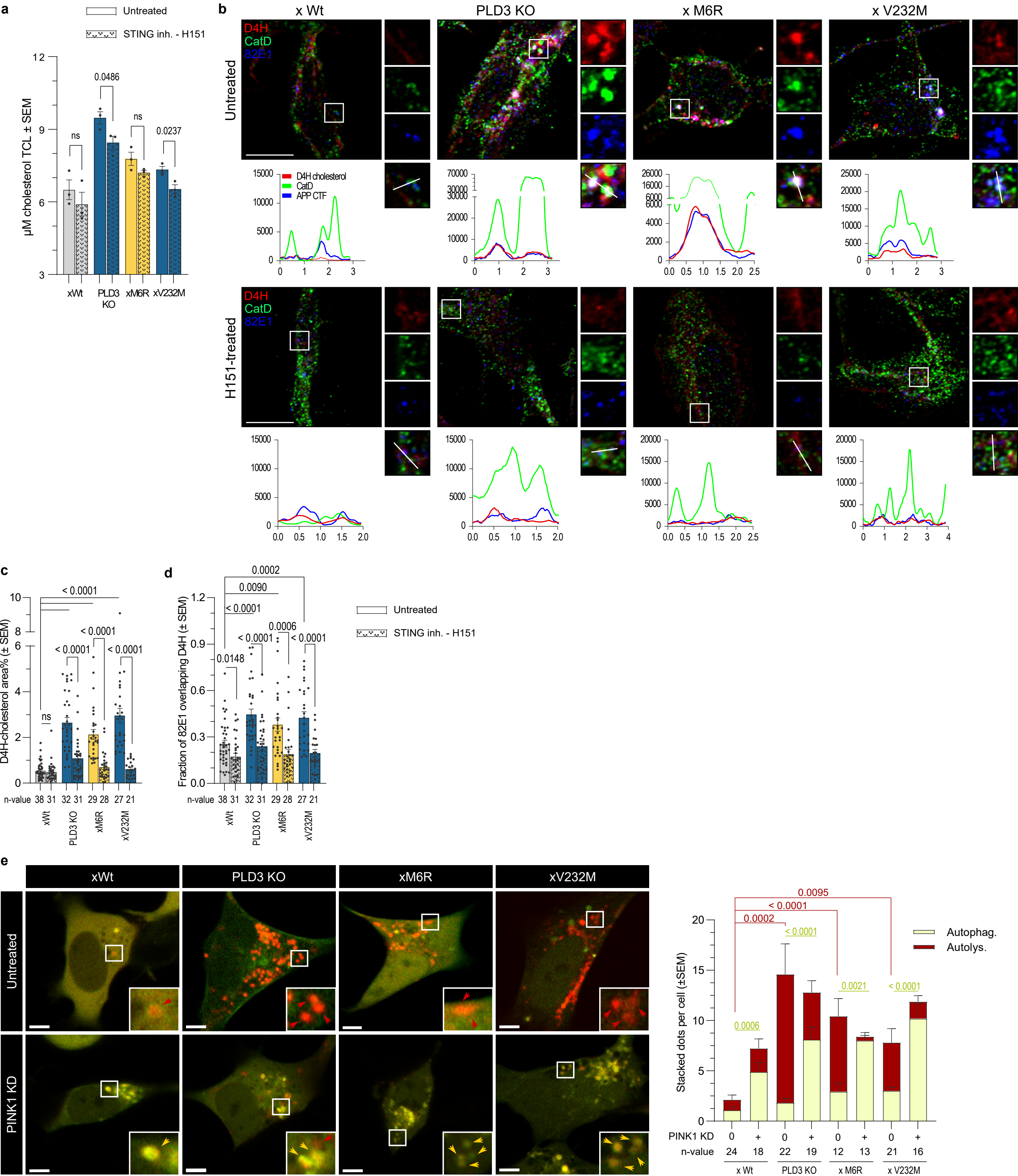
a
Untreated
STING inh. - H151
μM cholesterol TCL ± SEM
12
9
6
3
ns
0.0486
ns
0.0237
xWt
PLD3 KO
xM6R
xV232M
b
x Wt
PLD3 KO
x M6R
x V232M
Untreated
H151-treated
D4H
CatD
82E1
D4H cholesterol
CatD
APP CTF
c
< 0.0001
ns
< 0.0001
< 0.0001
< 0.0001
D4H-cholesterol area% (± SEM)
n-value 38 31 32 31 29 28 27 21
xWt
PLD3 KO
xM6R
xV232M
d
0.0002
0.0090
< 0.0001
0.0006
0.0148
< 0.0001
< 0.0001
Fraction of 82E1 overlapping D4H (± SEM)
n-value 38 31 32 31 29 28 27 21
xWt
PLD3 KO
xM6R
xV232M
Untreated
STING inh. - H151
e
xWt
PLD3 KO
xM6R
xV232M
Untreated
PINK1 KD
0.0095
< 0.0001
0.0002
< 0.0001
0.0006
0.0021
< 0.0001
Autophag.
Autolys.
Stacked dots per cell (±SEM)
PINK1 KD 0 + 0 + 0 + 0 +
n-value 24 18 22 19 12 13 21 16
x Wt
PLD3 KO
x M6R
x V232M

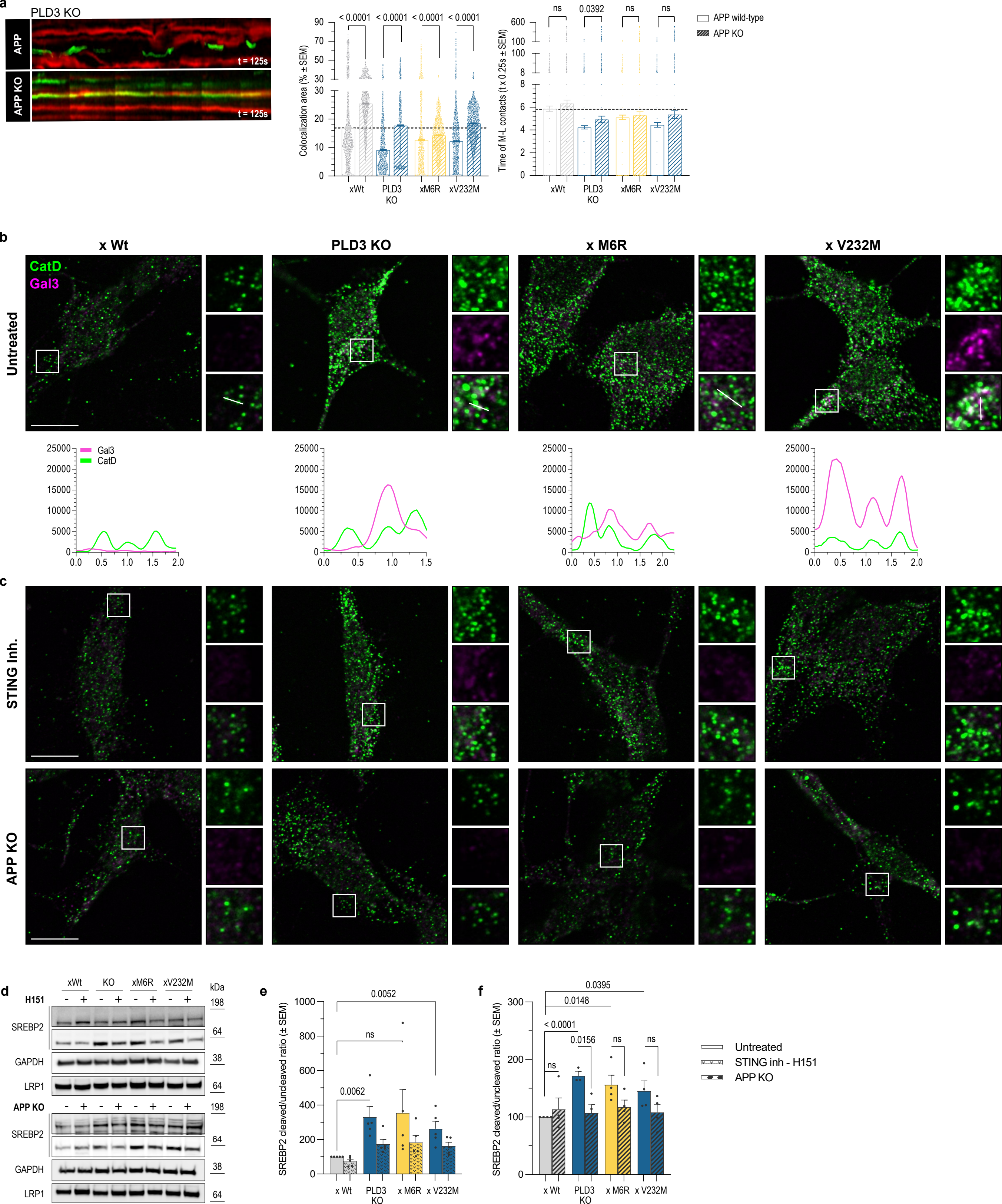
a
PLD3 KO
APP
APP KO
t = 125s
t = 125s
< 0.0001
< 0.0001
< 0.0001
< 0.0001
Colocalization area (% ± SEM)
xWt
PLD3 KO
xM6R
xV232M
ns
0.0392
ns
ns
Time of M-L contacts (t x 0.25s ± SEM)
APP wild-type
APP KO
b
x Wt
PLD3 KO
x M6R
x V232M
CatD
Gal3
Untreated
c
STING Inh.
APP KO
d
xWt
KO
xM6R
xV232M
kDa
H151
SREBP2
GAPDH
LRP1
APP KO
198
64
38
64
e
SREBP2 cleaved/uncleaved ratio (± SEM)
0.0052
ns
0.0062
x Wt
PLD3 KO
x M6R
x V232M
f
0.0395
0.0148
< 0.0001
0.0156
ns
ns
ns
Untreated
STING inh - H151
APP KO
