# Supplementary material for: Phospholipase D3 degrades mitochondrial DNA to regulate nucleotide signaling and APP metabolism
Source: Nat Commun. 2023 May 24;14:2847. doi: 10.1038/s41467-023-38501-w (PMC10209153; doi:10.1038/s41467-023-38501-w)
Supplement: Supplementary file 8 — Reporting Summary [file 41467_2023_38501_MOESM8_ESM.pdf]

nature portfolio

Corresponding author(s): Wim Annaert

Last updated by author(s): Apr 17, 2023

# Reporting Summary

Nature Portfolio wishes to improve the reproducibility of the work that we publish. This form provides structure for consistency and transparency in reporting. For further information on Nature Portfolio policies, see our Editorial Policies and the Editorial Policy Checklist.

## Statistics

For all statistical analyses, confirm that the following items are present in the figure legend, table legend, main text, or Methods section.

| n/a | Confirmed | |
|---|---|---|
| ☐ | ☒ | The exact sample size ($n$) for each experimental group/condition, given as a discrete number and unit of measurement |
| ☐ | ☒ | A statement on whether measurements were taken from distinct samples or whether the same sample was measured repeatedly |
| ☐ | ☒ | The statistical test(s) used AND whether they are one- or two-sided<br>*Only common tests should be described solely by name; describe more complex techniques in the Methods section.* |
| ☒ | ☐ | A description of all covariates tested |
| ☐ | ☒ | A description of any assumptions or corrections, such as tests of normality and adjustment for multiple comparisons |
| ☐ | ☒ | A full description of the statistical parameters including central tendency (e.g. means) or other basic estimates (e.g. regression coefficient) AND variation (e.g. standard deviation) or associated estimates of uncertainty (e.g. confidence intervals) |
| ☐ | ☒ | For null hypothesis testing, the test statistic (e.g. $F$, $t$, $r$) with confidence intervals, effect sizes, degrees of freedom and $P$ value noted<br>*Give P values as exact values whenever suitable.* |
| ☒ | ☐ | For Bayesian analysis, information on the choice of priors and Markov chain Monte Carlo settings |
| ☒ | ☐ | For hierarchical and complex designs, identification of the appropriate level for tests and full reporting of outcomes |
| ☒ | ☐ | Estimates of effect sizes (e.g. Cohen's $d$, Pearson's $r$), indicating how they were calculated |

*Our web collection on statistics for biologists contains articles on many of the points above.*

## Software and code

Policy information about availability of computer code

| | |
|---|---|
| Data collection | 1. CellR Software (Olympus) on an Olympus IX81 with an UAPO/340 40x oil objective (1.35 NA) - calcium imaging.<br>2. ZEN black (version 2.3, Carl Zeiss Microscopy GmbH) connected to an inverted Zeiss LSM microscope - live/fixed imaging.<br>3. LightCycler 480 Software (v1.5, Roche) - qPCR.<br>4. Agilent 2100 Expert Software (Agilent Technologies) connected to the Agilent 2100 Bioanalyzer System - electropherograms.<br>5. MultiQuantTM software version 3.0.3. - lipidomic peak integration.<br>6. Python Molmass 2019.1.1 - lipidomic corrections for isotopic contributions. |
| Data analysis | 1. Image J Software (Schindelin et al., 2012; Schneider et al., 2012) for immunofluorescent image analysis.<br>2. RADIUS 2.0 (Build 14402, Emsis) software for EM analysis of EL-mitochondrion contacts.<br>3. FlowJo (v10; Treestar, Ashland, OR, USA) for FC analysis.<br>4. Prism GraphPad (v9.2.0; La Jolla, CA, USA) for statistical analysis and visual representation.<br>5. Seahorse XFp Wave Software (Version 2.6, Agilent Technologies, Santa Clara, CA, USA) for mitochondrial fitness analysis. |

For manuscripts utilizing custom algorithms or software that are central to the research but not yet described in published literature, software must be made available to editors and reviewers. We strongly encourage code deposition in a community repository (e.g. GitHub). See the Nature Portfolio guidelines for submitting code & software for further information.

## Data

Policy information about availability of data

All manuscripts must include a data availability statement. This statement should provide the following information, where applicable:

- Accession codes, unique identifiers, or web links for publicly available datasets
- A description of any restrictions on data availability
- For clinical datasets or third party data, please ensure that the statement adheres to our policy

All data are available in the main text or the supplementary materials. Raw lipidomic data as well as an excel with all calculated values have been made available at the NIH Common Fund's National Metabolomics Data Repository (NMDR) website, the Metabolomics Workbench, https://www.metabolomicsworkbench.org where it has been assigned Study ID ST002334. The data can be accessed directly via its Project DOI: http://dx.doi.org/10.21228/M8740F. To whom correspondence should be addressed: Prof. Wim Annaert (e-mail: wim.annaert@kuleuven.be). Source data are provided with this paper.

## Human research participants

Policy information about studies involving human research participants and Sex and Gender in Research.

| | |
|---|---|
| Reporting on sex and gender | Not applicable |
| Population characteristics | Not applicable |
| Recruitment | Not applicable |
| Ethics oversight | Not applicable |

Note that full information on the approval of the study protocol must also be provided in the manuscript.

# Field-specific reporting

Please select the one below that is the best fit for your research. If you are not sure, read the appropriate sections before making your selection.

[x] Life sciences [ ] Behavioural & social sciences [ ] Ecological, evolutionary & environmental sciences

For a reference copy of the document with all sections, see nature.com/documents/nr-reporting-summary-flat.pdf

# Life sciences study design

All studies must disclose on these points even when the disclosure is negative.

| | |
|---|---|
| Sample size | No statistical methods were used to predetermine the sample size; n-values were based on our previous studies (PMID: 34292306, PMID: 28134274, PMID: 27293189), including omics, microscopy and WB. |
| Data exclusions | No data was excluded. |
| Replication | For all experiments, data are reported based on individual cells or biological replicates pooled from multiple cells, e.g. lysates. Individual cell values were obtained from at least 2 independent dishes/coverslips. For the TEM image analysis of Fig. 2e-f, two independent sample preps were performed: one on each PLD3 knockout (x rescue) clonal line (i.e. exon5 and exon6). Images of both clonal lines were pooled. Western blots are representative of two or more replicates (as indicated in analysis graphs and the source data file), with the exception of Fig. S1i. Lysosomal extractions on all SNPs in parallel was performed once. Statistical analysis was performed as from n=3. |
| Randomization | The samples were grouped based on their genotype or treatment condition. By keeping all samples in the same growth and medium conditions, covariates were minimized. For lipidomics, all sample conditions were examined in a single run. |
| Blinding | In this study, the investigators were not blinded, which was not possible for cell-based experiments. |

# Reporting for specific materials, systems and methods

We require information from authors about some types of materials, experimental systems and methods used in many studies. Here, indicate whether each material, system or method listed is relevant to your study. If you are not sure if a list item applies to your research, read the appropriate section before selecting a response.

## Materials & experimental systems

| n/a | Involved in the study |
| --- | --- |
| | ☒ Antibodies |
| | ☒ Eukaryotic cell lines |
| ☒ | ☐ Palaeontology and archaeology |
| | ☒ Animals and other organisms |
| ☒ | ☐ Clinical data |
| ☒ | ☐ Dual use research of concern |

## Methods

| n/a | Involved in the study |
| --- | --- |
| ☒ | ☐ ChIP-seq |
| | ☒ Flow cytometry |
| ☒ | ☐ MRI-based neuroimaging |

# Antibodies

Antibodies used

rabbit anti-APP (Y188, Ab32136, Abcam, 1:10.000 for WB, 1:500 for IF)
rabbit anti-ATG5 (2630, Cell Signaling Technology, 1:500 for WB)
rabbit anti-Cathepsin D (ab75852, Abcam, 1:1000 for WB, 1:100 for IF)
rabbit anti-LC3 B (NB600-1384, Novus Biologicals, 1:1000 for WB, 1:100 for IF)
rabbit anti-Lrp1 (ab92544, Abcam, 1:1000 for WB)
rabbit anti-PINK1 (BC100-494, Novus Biologicals, 1:1000 for WB)
rabbit anti-N-terminal PLD3 (in-house generated PMID: 33288674, 1:1000 for WB)
rabbit anti-C-terminal PLD3 (in-house generated this paper, 1:1000 for WB) - see validation
rabbit anti-SREBP2 (10007663, Cayman Chemical, 1:1000 for WB)
rabbit anti-STING (D1V5L, 50494S, Cell signaling, 1:500 for WB, 1:50 for IF)
rabbit anti-STING-P at Ser366 (D7C3S, 19781S, Cell signaling, 1:500 for WB)
rabbit anti-TBK1/NAK (D1B4, 3504S, Cell signaling, 1:500 for WB)
rabbit anti-TBK1/NAK at Ser172 (D52C2, 5483S, Cell signaling, 1:500 for WB)
rabbit anti-TLR9 (PA5-20203, Thermo Fisher Scientific, 1:250 for IF)

mouse anti-APP-CTF (82E1, IBL Co., 1:100 for IF)
mouse anti-cytochrome C (556433, BD Biosciences, 1:1000 for WB)
mouse anti-GAPDH (MAB374, EMD Millipore, 1:10.000 for WB)
mouse anti-IFN-α-R670 (APC, 130-099-214, Miltenyi Biotec, 1:10 for FC)
mouse anti-LAMP1 (611042, BD Biosciences, 1:500 for WB)
mouse anti-LAMP1 (CD107a, H4A3, BioLegend, 1:200 for IF)
mouse anti-GM130 (610823, BD Biosciences, 1:50 for IF)
mouse anti-Na+K+ ATPase alpha (#NB300-146, Novus Biologicals, 1:2000 for WB)
mouse anti-Rab7 (ab50533, Abcam, 1:500 for WB)
mouse anti-TNF-α-VS25 (BV510, 502949, Biolegend, 1:10 for FC)

rat-anti CD289-Y585 (TLR9-PE, eB72-1665, eBioscience, 1:200 for FC),
rat anti-galectin-3 (M3/38, Sc-23938, Santa Cruz, 1:100 for IF)
rat anti-Lamp1 (1D4B sc19992, Santa Cruz, 1:50 for IF)

goat anti-VPS35 (Ab10099, Abcam, 1:250 for IF)

Validation

The PLD3 C-terminal antibody was generated by immunization of rabbits with the human PLD3 C-terminal peptide WDSPYSHDLDTSADSVGNAC (Eurogentec). The peptide fused to keyhole limpet hemocyanin using the Imject Maleimide Activated Carrier Protein Spin Kit (Thermo Scientific), according to the manufacturer's instructions. New Zealand white rabbits were boosted 4 times at 1-month time intervals (P191/2017, Ethics Committee KU Leuven). The resulting sera were peptide-affinity purified on NHS Sepharose fast flow 75% slurry (Cytiva) and concentrated with Vivaspin 15R centrifugal tubes (25 min spin at 3000xg, VWR). Resulting purified antibody was aliquoted with an equal amount of glycerol and stored at -20°C until use.
Serum and purified antibodies were assessed for specificity on blot by validating band-specificity on total cell lysates and lysosomal extracts of wild-type and PLD3 KO SH-SY5Y cells.

Other antibodies were chosen from previous publications and/or validation by the manufacturers for their intended use. Where possible, we examined the antibodies' specificity, checking the target's known cellular localization, test their known responsiveness to stimuli and/or used knockdown or knockout cell lines.

# Eukaryotic cell lines

Policy information about cell lines and Sex and Gender in Research

Cell line source(s)

- The Alt-R CRISPR-Cas9 technology from IDTDNA was used for generation of PLD3 and APP knockout cell lines in the SH-SY5Y (ATCC, CRL-2266) background.
- We used the HEK293T (CRL-3216, ATCC) for lentiviral production.

Authentication

- Authentication of the SH-SY5Y cells was performed by DNA-STR-Typing at the Leibniz-Institut on 19.02.2016 (No.: A1602030-1).

| | |
|---|---|
| | - No additional authentication was performed on the HEK293T cells. Of note, these cells were only used for virus generation and not for biological/cell analyses. |
| Mycoplasma contamination | All cell lines are tested every 2 months according to the center's SOP. All cells were mycoplasma negative. |
| Commonly misidentified lines (See ICLAC register) | No misidentified lines were used in the study. |

## Animals and other research organisms

Policy information about studies involving animals; ARRIVE guidelines recommended for reporting animal research, and Sex and Gender in Research

| | |
|---|---|
| Laboratory animals | The polyclonal PLD3 antibody was generated in adult, female New Zealand White rabbits. |
| Wild animals | The study did not involve wild animals. |
| Reporting on sex | Rabbits were solely used for the purpose of generating antibodies. No biological information was obtained. |
| Field-collected samples | The study did not involve samples collected from the field. |
| Ethics oversight | The experiments were approved by the KULeuven Ethical Committee for Animal Experimentation and designated as project 191/2017. The animal facilities have an NIH animal welfare assurance (no #A5435-01). |

Note that full information on the approval of the study protocol must also be provided in the manuscript.

## Flow Cytometry

### Plots

Confirm that:

☒ The axis labels state the marker and fluorochrome used (e.g. CD4-FITC).

☒ The axis scales are clearly visible. Include numbers along axes only for bottom left plot of group (a 'group' is an analysis of identical markers).

☒ All plots are contour plots with outliers or pseudocolor plots.

☒ A numerical value for number of cells or percentage (with statistics) is provided.

### Methodology

| | |
|---|---|
| Sample preparation | Flow cytometry<br>All flow cytometric analyses were performed on a BD Fortessa X-20 analyzer and the stopping gate was set at 10.000 events. Data was analyzed using FlowJo (v10; Treestar, Ashland, OR, USA). The FSC-A/SSC-A plot was used to eliminate debris and the FSC-H/FSC-A dot plot for identifying singlets.<br>MitoProbe JC1 assay. 1E06 cells were incubated with 2 µM JC1 mitochondrial membrane potential probe (T3168, Thermo Fisher Scientific) for 30 minutes at 37°C (5% CO2) before analysis. JC1 responsiveness to membrane potential alterations was validated with carbonyl cyanide 3-chlorophenylhydrazone (CCCP, C2759, Sigma-Aldrich)-mediated mitochondrial depolarization (6h, 30 µM). Cell populations were plotted in contour plots and the percentage of mitochondrial depolarization indicated.<br>Intracellular staining. Protein secretion was inhibited in 1E06 cells by incubation with monensin (2µM, 00-4505-51, Invitrogen) for 3h at 37°C. Intracellular Staining was performed following the Foxp3 Fixation/Permeabilization buffer set protocol (00-5523-00, eBioscience) and listed antibodies. mean fluorescent intensity (MFI) values were calculated using FlowJo.<br>Lysosomal permeability. 1E06 cells were stained with 50 nm of lysosomotropic dye (LysoTracker Red DND-99, L7528, Thermo Fisher Scientific) and LIVE/DEAD™ Fixable Green Dead Cell Stain (L34969, Thermo Fischer Scientific) for 15 min. Positive control conditions were pre-incubated with 1 mM LLOMe (L7393, Sigma) for 10 min at 37°C and STING inhibition conditions with 1 µM H-151 (941987-60-6, Sanbio) for 5h. The MFI was calculated and the leakiness propensity calculated as the +LLOME/untreated ratio. For Gal3 staining, see confocal microscopy section.<br>Lysosomal function according to the DQ-Red BSA lysosomal protease activity assay (D12051, Invitrogen). Cells were incubated with 10 µg/ml DQ Red BSA and 50 nM LysoTracker green for 30 minutes at 37°C. After two wash steps, cells were analyzed at four time points (0, 30, 60, 90 min), allowing for kinetic slope calculation. Activities were analyzed relative to the LysoTracker signal. |
| Instrument | All flow cytometric analyses were performed on a BD Fortessa X-20 analyzer. |
| Software | Data was analyzed using FlowJo (v10; Treestar, Ashland, OR, USA) |
| Cell population abundance | Samples were not sorted, only analysed by flow cytometry. |
| Gating strategy | The gating strategy was added to the (supplementary) figures. |

☒ Tick this box to confirm that a figure exemplifying the gating strategy is provided in the Supplementary Information.
